# Cardiometabolic factors and risk of dementia in people with type 2 diabetes in England: a large retrospective cohort study

**DOI:** 10.1136/bmjdrc-2025-005743

**Published:** 2026-08-17

**Authors:** Heidi TM Lai, Kiara Chang, Mansour TA Sharabiani, Jonathan Valabhji, Edward W Gregg, Lefkos T Middleton, Azeem Majeed, Jonathan Pearson-Stuttard, Christopher J Millett, Alex Bottle, Eszter P Vamos

**Affiliations:** 1 Public Health Policy Evaluation Unit, School of Public Health, Imperial College London, London, England, UK; 2 Department of Primary Care and Public Health, School of Public Health, Imperial College London, London, England, UK; 3 Department of Metabolism, Digestion and Reproduction, Faculty of Medicine, Chelsea and Westminster Campus, Imperial College London, London, England, UK; 4 School of Population Health, RCSI University of Medicine and Health Sciences, Dublin, Leinster, Ireland; 5 Department of Epidemiology and Biostatistics, School of Public Health, Imperial College London, London, England, UK; 6 Imperial College Healthcare NHS Trust, London, England, UK; 7 Ageing Epidemiology Research Unit, School of Public Health, Imperial College London, London, England, UK; 8 Health Analytics, Lane Clark & Peacock LLP, London, England, UK; 9 Northumbria Healthcare NHS Foundation Trust, North Shields, Newcastle upon Tyne, England, UK; 10 NOVA National School of Public Health, Public Health Research Centre, Comprehensive Health Research Center, CHRC, NOVA University Lisbon, Lisbon, Lisbon, Portugal

**Keywords:** Diabetes Mellitus, Type 2, Dementia, Blood Pressure, Body Mass Index

## Abstract

**Introduction:**

People with type 2 diabetes (T2D) have a higher dementia risk. Cardiometabolic factors may influence dementia development, but long-term links among people with T2D remain unclear. Prior studies show mixed findings and have not considered how the time before dementia diagnosis at which cardiometabolic factors are measured affects their association with dementia risk.

**Methods:**

We examined the associations between eight routinely measured cardiometabolic factors (including blood pressure, lipid, and glycemic markers) and the risk of dementia in 249 958 adults with T2D from the Clinical Practice Research Datalink (1999–2018). Multivariable Cox proportional hazards regression was applied with age as the timescale and stratified by follow-up time at 5, 5–10, and ≥10 years before dementia diagnosis. Cardiometabolic factors were analyzed continuously and categorically using clinically relevant cut-offs.

**Results:**

22 492 incident dementia cases were observed (1 257 998 person-years of follow-up). Higher systolic blood pressure was associated with a lower dementia risk, particularly when measured closer to the time of dementia diagnosis. Higher diastolic blood pressure (DBP) (90 vs 80 mm Hg) was associated with a 21% greater risk of dementia (95% CI 15% to 27%). Dementia risk was 32% (27%–36%) lower for those with a body mass index (BMI) ≥30 (vs <25 kg/m^2^) when measured within 5 years of dementia diagnosis, but 15% (7%–23%) and 43% (31%–56%) higher if measured 5–10 and ≥10 years before diagnosis, respectively. Higher levels of glycemic measures were consistently associated with a greater dementia risk. Findings for total cholesterol and low-density lipoprotein were largely null.

**Conclusions:**

Higher DBP, BMI, and glycemic measures measured over 10 years before dementia diagnosis were linked to a greater dementia risk among adults with T2D. Associations varied depending on when cardiometabolic factors were measured relative to dementia diagnosis, demonstrating the importance of distinguishing potential long-term risk factors from short-term markers of dementia development.

**WHAT IS ALREADY KNOWN ON THIS TOPIC**

**WHAT THIS STUDY ADDS**

- Higher diastolic blood pressure, body mass index, and glycemic markers measured ≥10 years before dementia diagnosis were associated with greater long-term dementia risk among people with type 2 diabetes. However, when measured closer to diagnosis, these associations reversed or attenuated. Lipid markers (total cholesterol and low-density lipoprotein) showed largely null or short-term associations, while high-density lipoprotein was protective only when measured ≥10 years before diagnosis.

**HOW THIS STUDY MIGHT AFFECT RESEARCH, PRACTICE, OR POLICY**

- The results suggest that late-life declines in blood pressure, body mass index, and glycemic measures may serve as markers of emerging cognitive decline and could inform earlier identification strategies in primary care. Future research should consider the timing of cardiometabolic measurements to distinguish short-term associations that may reflect dementia development (potentially indicating reverse causation) from long-term effects that may represent risk or protective factors.

## Introduction

Dementia is the leading cause of death in the UK, with 11.4% of deaths in England and Wales attributable to dementia in 2022.^1^ Type 2 diabetes (T2D) is a major risk factor for dementia.^2^ Although the complex mechanisms linking these conditions are still being elucidated, coexisting modifiable risk factors such as hypertension, obesity, and dyslipidemia may contribute to dementia risk in people with T2D.^2^ Cerebral insulin resistance, vascular endothelial dysfunction, inflammation, and blood–brain barrier injury have also been implicated in increased dementia risk.^3^ However, the relationship between modifiable cardiometabolic risk factors and dementia risk in individuals with T2D is generally mixed.^4–10^ Additionally, most studies are cross-sectional, often lacking substantive follow-up with limited adjustment for potential confounders.^5–10^

In the general population, cardiometabolic factors have been linked to dementia risk, but findings remain inconsistent.^11–13^ One possible explanation is the challenge of accounting for the long preclinical phase that precedes dementia diagnosis. One previous study demonstrated that the observed association between higher body mass index (BMI) and reduced dementia risk in late life might be confounded by substantial weight loss during preclinical dementia.^12^ Consequently, while higher midlife BMI has been linked to an increased late-life dementia risk, associations based on late-life BMI are less clear and may reflect weight loss occurring before diagnosis.^12^ We also demonstrated a declining trajectory in mean systolic blood pressure (SBP) and BMI over 20 years of follow-up, where reductions were more pronounced in people with T2D who later developed dementia versus those who did not.^14^ This alludes to the possibility of reverse causation between late-life cardiometabolic factors and dementia,^14^ potentially impacting disease progression differently across the lifespan.^3^ In other words, the relationship between cardiometabolic factors and imminent dementia risk may differ from that with distal risks of dementia. Changes in cardiometabolic factors may indicate preclinical dementia development, rather than serving as risk factors,^12^ making the timing of assessing cardiometabolic factors as exposures critically important.

As previous studies did not consider how the influence of cardiometabolic factors varies at different time points relative to dementia diagnosis, we aimed to address this by (1) examining associations between eight routinely measured cardiometabolic factors and dementia risk in a large cohort of adults with T2D and (2) characterizing dementia risk by stratifying the analyses according to the length of follow-up.

## Methods

### Study design

The UK Clinical Practice Research Datalink (CPRD) ‘GOLD’ holds longitudinal pseudonymized primary care electronic health records.^15^ In this extraction, a subset of 410 English practices was linked to Hospital Episode Statistics (HES) and Office for National Statistics (ONS) mortality files and covers approximately 7% of the English population. Sociodemographic characteristics, clinical history and assessments, laboratory tests, and prescriptions were collected prospectively during routine care for each individual.^15^ A total of 249 958 people aged ≥45 years with T2D at baseline were included in the final sample (online supplemental eFigure 1).

### T2D ascertainment

T2D was ascertained using diagnostic (C10) and management (66A) ‘Read codes’, prescription data for glucose-lowering therapy, and International Classification of Diseases, 10th revision diagnostic codes in HES and ONS records as previously published.^14^ Individuals who were diagnosed with T2D at any time between January 1, 1999, and December 31, 2018, and met CPRD quality assurance criteria for research use^15^ were considered for inclusion. Possible type 1 diabetes cases were excluded, as described previously.^14^

### Study exposures

SBP, diastolic blood pressure (DBP), BMI, fasting plasma glucose (FPG), hemoglobin A1c (HbA1c), total cholesterol, high-density lipoprotein (HDL), and low-density lipoprotein (LDL) measurements were averaged as annual means per individual. Annual ascertainment is reasonably robust, as the management of chronic diseases and recording of care processes have been financially incentivized in English primary care since 2004.^16^

For participants who were diagnosed with T2D before the index date (January 1, 2003), the closest recorded annual measure of cardiometabolic factors to the index date was considered as baseline. Otherwise, baseline cardiometabolic levels were determined in the year of T2D diagnosis. This lookback period is essential because dementia, comorbidities, and cardiometabolic factors are often underrecorded if only same-year data are used. Blood pressure and glycemic measures were first investigated continuously, then assessed categorically with three groups (or two groups for lipids) using clinically meaningful thresholds^17^: <130, 130–139, and ≥140 mm Hg (SBP); <80, 80–89, and ≥90 mm Hg (DBP); <25.0, 25.0–29.9, and ≥30.0 kg/m^2^ (BMI); <7.0, 7.08.5, and >8.5 mmol/L (FPG); <48, 48–58, and >58 mmol/mol (HbA1c); ≤5.0 and >5.0 mmol/L (total cholesterol); <1.0 and ≥1.0 mmol/L (HDL); and ≤3.0 and >3.0 mmol/L (LDL).

### Outcome

Incident dementia was identified using a previously published algorithm,^14^ which included several data sources (including cognitive testing) to maximize the detection of dementia due to underrecording in electronic health records, including combinations of clinical diagnostic and administrative codes, cognitive functioning testing, prescription of dementia drugs, referrals, and diagnostic codes on hospital admissions and mortality data. After taking into account the data sources listed, if individuals had a cognitive test recorded without a valid result, they were classified as a ‘probable case’ if they also had diagnostic and administrative management codes that refer to mild/moderate cognitive impairment, memory loss, or referral to a memory clinic in combination. To further maximize case detection for sensitivity analyses, we also identified ‘possible dementia cases’ defined as having a referral to a memory clinic (n=189), which increased the total number of dementia cases to 22 681.

### Definition of covariates

Age is defined by date of birth and sex as male or female, and indeterminate gender (n=5) was excluded. Ethnicity (White, non-White, and missing) was determined from the combination of CPRD and HES records. If ethnicity could not be determined from either source, they were assigned as ‘missing/unknown’ (6%). Smoking status (people who smoke, people who had previously smoked, and people who do not smoke) was defined using Read codes recorded closest to the baseline. The Index of Multiple Deprivation (IMD)^18^ was provided in quintiles. The duration of diabetes at baseline was determined from the date of the first diagnosis of T2D. Comorbid conditions at baseline were defined as the total number of comorbidities, including stroke, acute myocardial infarction, peripheral artery disease, atrial fibrillation, heart failure, asthma, chronic lung disease, malignancies, chronic kidney disease, retinopathy, rheumatoid arthritis, Parkinson’s disease, and depression, all defined based on individuals’ history from a combination of primary and secondary care data.

### Statistical analysis

Cox proportional hazards modeling (using age as the timescale)^19^ evaluated the association between baseline levels of cardiometabolic factors and incident dementia in the full and subcohorts, stratified by follow-up time (<5 years, 5–10 years, and ≥10 years). All models in the main analyses were adjusted for previously listed covariates. Individuals contributed person-time from the index date of January 1, 2003, or date of incident T2D diagnosis thereafter until dementia diagnosis, death, transfer out of practice, or end of study period on December 31, 2018, whichever occurred first. Kaplan-Meier survival curves, log cumulative hazard functions, and Schoenfeld residuals provided no evidence of proportional hazards assumption violation.

Each cardiometabolic factor on incident dementia risk was modeled continuously using restricted cubic splines with Harrell knots and categorically by groups based on clinically relevant thresholds reported above.^20^ For restricted cubic splines, likelihood ratio tests guided the number of knots by comparing the default minimum of three knots with four knots or more. The most parsimonious model was prioritized.

We conducted several sensitivity analyses. The inclusion of possible dementia cases tested the robustness of our dementia definition. Additional adjustments for baseline medication prescription (binary variables) use were examined, including antihyperglycemic (defined as insulin and oral antihyperglycemic medications), antihypertensive, antilipid, and antiplatelet medications, which reflect clinical management rather than underlying biological levels of risk factors. Furthermore, to ensure the regression estimates were not biased by the systematic differences in cohort characteristics between subcohorts with different follow-up times (eg, those with shorter follow-up times were comparatively older), inverse-probability weighting was applied in a sensitivity analysis to adjust the balance of study covariates according to the distribution in the overall cohort (all model covariates, including medications). Lastly, to account for death as a competing risk and to take into account the fact that those who died could have developed dementia if they had lived longer, we repeated the analyses using a modified Fine and Gray model.^21^

Logistic regression calculated the odds of being included in the subcohort with respect to the full cohort. Model covariates include baseline age, sex, ethnicity, Index of Multiple Deprivation, smoking, duration of diabetes, comorbid conditions, antihypertension medication, antilipid medication, antihyperglycemic medication, insulin use, and antiplatelet medication. Inverse probability was calculated as 1/the odds of being included in the subcohort. With inverse probability weighting applied, all subcohorts were comparable to the full cohort (online supplemental eTable 1).

All statistical analyses were performed in Stata 17.0; a two-sided p<0.05 was considered statistically significant.

## Results

Among 249 958 people with T2D, 22 492 incident dementia cases were observed during 1 257 998 person-years of follow-up (table 1). Among individuals with <5 years of follow-up, 7.4% experienced incident dementia, while 10.0% and 13.3% experienced incident dementia for subcohorts with 5–10 years and ≥10 years of follow-up, respectively. Mean (SD) follow-up was 2.1 (1.4) years, 7.2 (1.4) years, and 12.4 (1.7) years for subcohorts with <5 years, 5–10 years, and ≥10 years of follow-up, respectively.

**Table 1:** Baseline participant characteristics in the Clinical Research Practice Datalink by follow-up period in England

Variables	Overall	Follow-up time			P value*
		<5 years	5–10 years	≥10 years	
Participants, n	249 958	143 257	71 075	35 626	
Age, years, mean (SD)	65.5 (11.7)	66.5 (12.4)	64.7 (11.0)	62.9 (9.6)	<0.001
Sex, n (%)					
Male	133 596 (53.4)	75 937 (53.0)	38 291 (53.9)	19 368 (54.4)	<0.001
Female	116 362 (46.6)	67 320 (47.0)	32 784 (46.1)	16 258 (45.6)	
Ethnicity, n (%)					
White	210 448 (84.2)	117 507 (82.0)	61 573 (86.6)	31 368 (88.1)	<0.001
Non-White	26 046 (10.4)	16 736 (11.7)	6379 (9.0)	2931 (8.2)	
Unknown/missing	13 464 (5.4)	9014 (6.3)	3123 (4.4)	1327 (3.7)	
Smoking status, n (%)					
People who do not smoke	94 580 (37.8)	57 046 (39.8)	25 247 (35.5)	12 287 (34.5)	<0.001
People who smoke	81 311 (32.5)	46 469 (32.4)	23 616 (33.2)	11 226 (31.5)	
People who had previously smoked	74 067 (29.6)	39 742 (27.7)	22 212 (31.3)	12 113 (34.0)	
Index of Multiple Deprivation quintiles, n (%)					
First quintile (least deprived)	34 822 (13.9)	19 880 (13.9)	9615 (13.5)	5327 (15.0)	<0.001
2	47 271 (18.9)	27 166 (19.0)	13 217 (18.6)	6888 (19.3)	
3	48 349 (19.3)	27 672 (19.3)	13 685 (19.3)	6992 (19.6)	
4	55 579 (22.2)	32 770 (22.9)	15 580 (21.9)	7229 (20.3)	
Fifth quintile (most deprived)	63 937 (25.6)	35 769 (25.0)	18 978 (26.7)	9190 (25.8)	
Diabetes duration, years, mean (SD)	1.67 (3.71)	1.87 (4.13)	1.30 (3.15)	1.62 (2.85)	<0.001
Dementia incidence, years, mean (SD)	22 492 (9.0)	10 662 (7.4)	7086 (10.0)	4744 (13.3)	
Follow-up duration, years, mean (SD)	5.03 (4.01)	2.12 (1.43)	7.21 (1.43)	12.4 (1.72)	<0.001
Medication and comorbidities					
Antihypertensive medication, n (%)	152 587 (61.0)	83 693 (58.4)	45 290 (63.7)	23 604 (66.3)	<0.001
Antilipid medication, n (%)	128 749 (51.5)	69 120 (48.3)	39 921 (56.2)	19 708 (55.3)	<0.001
Oral antihyperglycemic medication, n (%)	103 556 (41.4)	54 448 (38.0)	30 679 (43.2)	18 429 (51.7)	<0.001
Insulin, ever prescribed, n (%)	12 968 (5.2)	7877 (5.5)	3099 (4.4)	1992 (5.6)	<0.001
Antiplatelet medication, n (%)	64 811 (25.9)	34 410 (24.0)	20 224 (28.5)	10 177 (28.6)	<0.001
Number of comorbidities, median (IQR)†	0 (0–1)	0 (0–2)	0 (0–1)	0 (0–1)	<0.001
Cardiometabolic factors, mean (SD)					
Systolic blood pressure (mm Hg)	139 (16)	137 (16)	140 (15)	142 (16)	<0.001
Diastolic blood pressure (mm Hg)	79.3 (9.2)	78.5 (9.4)	80.0 (8.8)	81.1 (8.6)	<0.001
Body mass index (kg/m²)	30.5 (6.3)	30.3 (6.5)	30.7 (6.2)	30.6 (6.0)	<0.001
Fasting plasma glucose (mmol/L)	8.91 (3.80)	8.80 (3.90)	8.94 (3.71)	9.20 (3.63)	<0.001
Hemoglobin A1c (mmol/mol)	57.2 (17.3)	57.2 (18.0)	57.1 (16.8)	57.0 (15.8)	0.049
Total cholesterol (mmol/L)	4.89 (1.14)	4.85 (1.18)	4.92 (1.11)	5.0 (1.05)	<0.001
High-density lipoprotein (mmol/L)	1.27 (0.38)	1.27 (0.39)	1.26 (0.36)	1.28 (0.37)	<0.001
Low-density lipoprotein (mmol/L)	2.84 (0.99)	2.85 (1.03)	2.82 (0.96)	2.86 (0.93)	<0.001

* Two-sample independent t-test for continuous variables and chi² test for categorical variables were performed to compare groups stratified by follow-up time.

† Comorbidities include stroke, acute myocardial infarction, peripheral artery disease, atrial fibrillation, heart failure, asthma, chronic obstructive pulmonary disease, malignancies, chronic kidney disease, retinopathy, rheumatoid arthritis, Parkinson’s disease, and clinical depression.

### Participant baseline characteristics

At baseline, the full cohort mean age (SD) was 65 years^12^; 46.6% were female; 84.2% were White, and the mean duration of diabetes (SD) was 1.67 (3.71) years (table 1). Participants in the <5 years of follow-up subcohort tended to be older, female, with a higher proportion of individuals who are not White, people who do not smoke, and a longer duration of diabetes but a lower proportion in most deprived areas (IMD quintile 5) and medication prescriptions than those with longer follow-ups. Baseline levels of blood pressure (SBP and DBP), BMI, FPG, and total cholesterol were also lower in the group with <5 years of follow-up relative to other groups. Absolute differences in levels of HbA1c, HDL, and LDL were small across the subcohorts, though reaching statistical significance. Participant characteristics by the eight exposure categories are reported in online supplemental eTables 2–9.

### Blood pressure and risk of dementia

**Figure 1:**
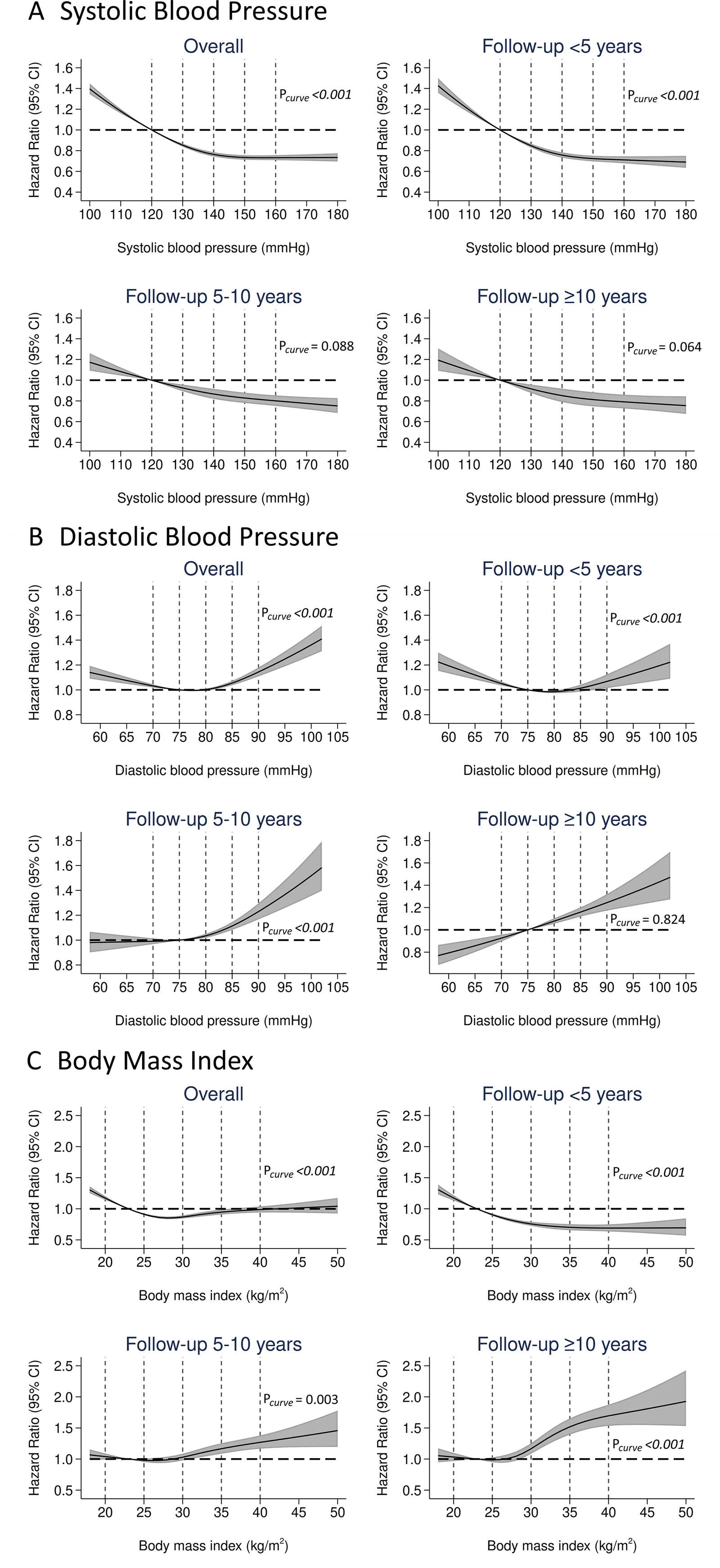
The association between (**A**) systolic blood pressure, (**B**) diastolic blood pressure, (**C**) body mass index, and risk of dementia among people with type 2 diabetes in the Clinical Practice Research Datalink in England, evaluated using restricted cubic splines and stratified by follow-up periods. The solid lines and shaded area represent the central risk estimate and 95% CI, respectively. The dotted vertical lines are reference lines, corresponding to (**A**) 120, 130, 140, 150, and 160 mm Hg, (**B**) 70, 75, 80, 85, and 90 mm Hg, and (**C**) 20, 25, 30, 35, and 40 kg/m^2^. The top and bottom 1% of participants were omitted as outliers to provide better visualization. Evidence for non-linearity (P_curve_) was calculated by performing a likelihood ratio test between a multivariable model with all spline terms versus a multivariable model with only the linear term. Model adjustments include sex, ethnicity, calendar year of entry, Index of Multiple Deprivation, smoking, total comorbidities, insulin use, and diabetes duration.

In the full cohort, SBP >140 mm Hg was associated with a lower risk of dementia, but SBP <140 mm Hg was associated with an increased risk of dementia (figure 1). This pattern was consistent in the subcohort with <5 years of follow-up, while those with 5–10 years and ≥10 years of follow-up showed a mild inverse association with dementia risk for SBP >140 mm Hg. Categorical analyses for SBP ≥140 mm Hg reported a 26% lower risk of dementia versus <130 mm Hg in the total cohort (online supplemental eTable 10). The inverse association was consistent across subcohorts and more pronounced in people with an SBP measurement within 5 years of dementia diagnosis (29%, 18%, and 20% lower risk of dementia among the ≥140 mm Hg vs the <130 mm Hg group, corresponding to subcohorts with <5, 5–10, and ≥10 years of follow-up, respectively).

In the overall cohort, the risk of dementia was lower for those with DBP between 75 and 80 mm Hg, suggesting a possible U-shaped association (figure 1). However, when DBP was measured 5–10 years before dementia diagnosis, a J-shaped association with dementia risk was evident, with an increased dementia risk over 75 mm Hg. Among individuals with ≥10 years of follow-up, a positive linear association was consistently observed. By categories, DBP within 80–89 mm Hg and ≥90 mm Hg was associated with a 4% and 21% higher risk of dementia in the total cohort, respectively, relative to DBP <80 mm Hg (online supplemental eTable 10). Subcohorts with longer follow-up time echoed these findings, ranging from 34% to 37% higher dementia risk in the 5–10 years and ≥10 years of follow-up groups, respectively, compared with the <5 years of follow-up group (online supplemental eTable 10).

### BMI and risk of dementia

In the full cohort, risks of dementia were similar among people with a BMI >35 kg/m^2^, while an inverse association was observed for BMI <28 kg/m^2^ (figure 1). Corresponding categorical analyses reported that BMI >30 kg/m^2^ was associated with a 10% lower risk of dementia versus BMI <25 kg/m^2^ (online supplemental eTable 10).

However, the associations of BMI and dementia risk differed considerably depending on the timing of BMI measurement. Among individuals with <5 years of follow-up, BMI was inversely associated with dementia for BMI values <35 kg/m^2^, whereas the risk of dementia remained lower thereafter (figure 1). Associations for subcohorts with 5–10 years and ≥10 years of follow-up were similar; BMI <27 kg/m^2^ was associated with a lower risk of dementia. Dementia risk among those with ≥30 kg/m^2^ was 32% lower in people with <5 years of follow-up but 15% and 43% higher with increasing follow-up time (5–10 years and ≥10 years), respectively, relative to BMI <25 kg/m^2^ (online supplemental eTable 10).

### Glycemic measures and risk of dementia

**Figure 2:**
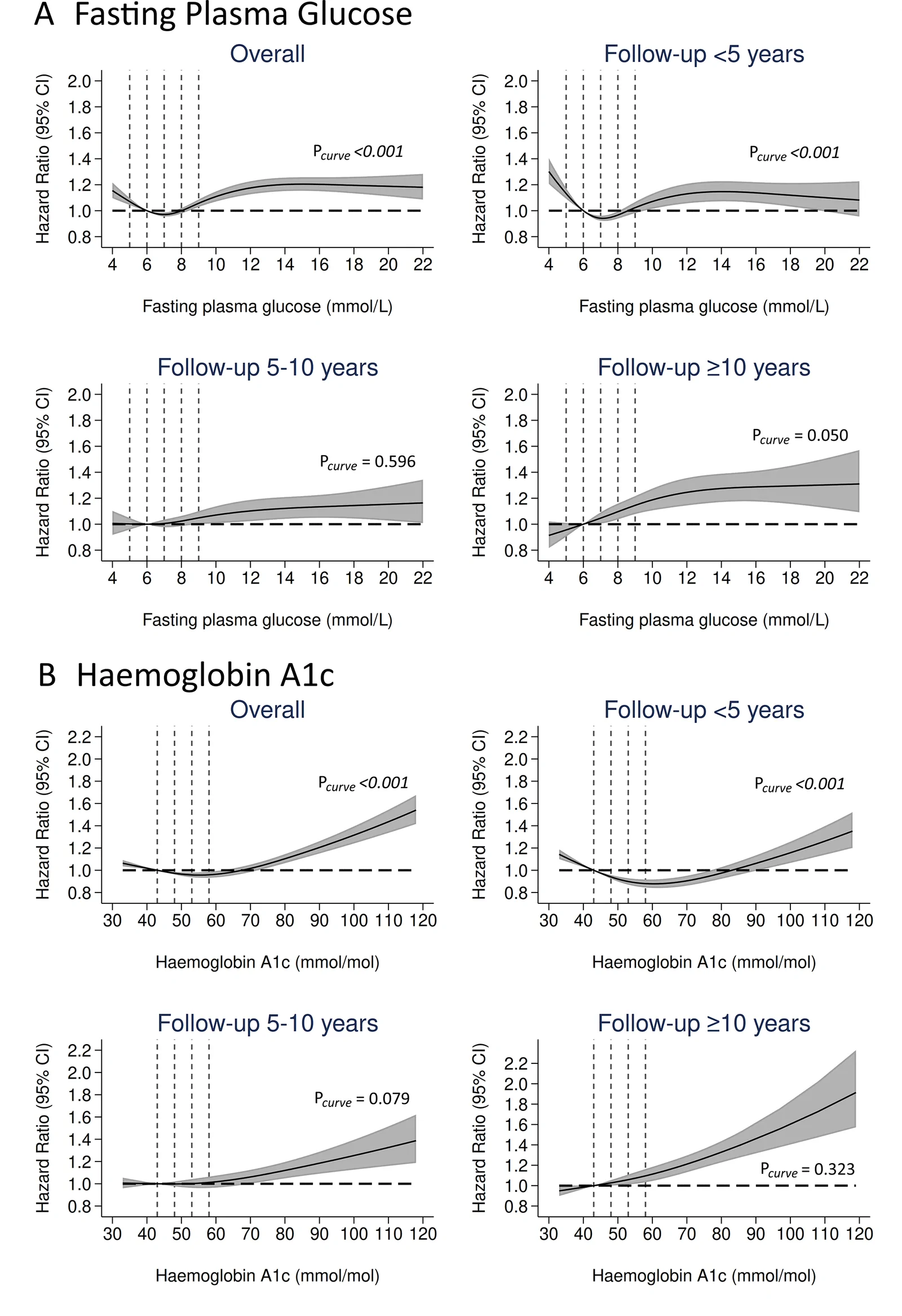
The association between (**A**) fasting plasma glucose, (**B**) hemoglobin A1c, and risk of dementia among people with type 2 diabetes in the Clinical Practice Research Datalink in England, evaluated using restricted cubic splines and stratified by follow-up periods**.** The solid lines and shaded area represent the central risk estimate and 95% CI, respectively. The dotted vertical lines are reference lines, corresponding to (**A**) 5, 6, 7, 8, and 9 mmol/L and (**B**) 43, 48, 53, and 58 mmol/mol. The top and bottom 1% of participants were omitted as outliers to provide better visualization. Evidence for non-linearity (P_curve_) was calculated by performing a likelihood ratio test between a multivariable model with all spline terms versus a multivariable model with only the linear term. Model adjustments include sex, ethnicity, calendar year of entry, Index of Multiple Deprivation, smoking, total comorbidities, insulin use, and diabetes duration.

Using splines, a U-shaped association was observed in the full cohort among those with an FPG <12 mmol/L, while those above had a similarly higher dementia risk (figure 2). Patterns of association were consistent across subcohorts, but the U-shaped association was only observed in those with <5 years of follow-up, whereas a positive linear association among FPG values <12 mmol/L was observed in those with longer follow-up time. In analyses using cut-offs, the FPG >8.5 mmol/L group (vs <7.0 mmol/L) reported a 13% higher risk of dementia in the full cohort (online supplemental eTable 10). Dementia risk for subcohorts with increasing follow-up time was found to be 6%, 8%, and 24% higher, respectively, for the FPG >8.5 mmol/L group versus <7.0 mmol/L (online supplemental eTable 10).

Findings for HbA1c showed a non-linear association with increasing risk of dementia (J-shaped curve) in the full cohort (figure 2), which was particularly apparent among the subcohort with <5 years of follow-up. Those with a longer follow-up time, however, were seen to have a monotonically increasing risk of dementia with higher HbA1c. By categories versus the <48 mmol/mol group in the full cohort, the 48–58 mmol/mol group had a 6% lower risk of dementia, while the >58 mmol/mol group had a 7% higher risk (online supplemental eTable 10). However, with longer follow-up, the association between higher HbA1c levels and greater risk of dementia strengthened (online supplemental eTable 10).

### Lipid levels and risk of dementia

**Figure 3:**
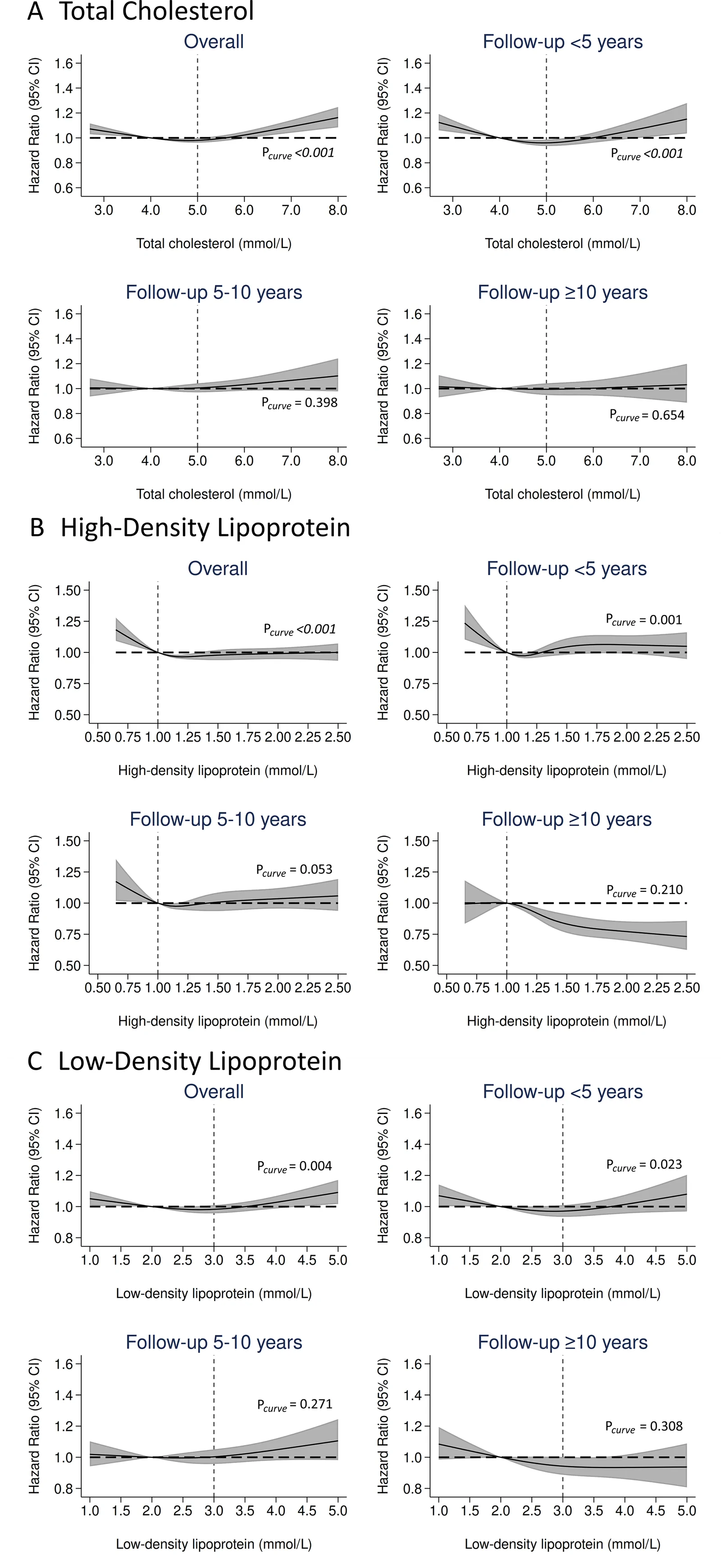
The association between (**A**) total cholesterol, (**B**) high-density lipoprotein, (**C**) low-density lipoprotein, and risk of dementia among people with type 2 diabetes in the Clinical Practice Research Datalink in England, evaluated using restricted cubic splines and stratified by follow-up periods. The solid lines and shaded area represent the central risk estimate and 95% CI, respectively. The dotted vertical lines are reference lines, corresponding to (**A**) 5 mmol/L, (**B**) 1 mmol/L, and (**C**) 3 mmol/L. The top and bottom 1% of participants were omitted as outliers to provide better visualization. Evidence for non-linearity (P_curve_) was calculated by performing a likelihood ratio test between a multivariable model with all spline terms versus a multivariable model with only the linear term. Model adjustments include sex, ethnicity, calendar year of entry, Index of Multiple Deprivation, smoking, total comorbidities, insulin use, and diabetes duration.

In the full cohort, higher levels of total cholesterol >5.0 mmol/L and LDL >3.0 mmol/L were non-linearly associated with a greater risk of dementia, but this was attenuated with an increasing length of follow-up time (figure 3). No significant association was found when assessing dementia risk by analysis using cut-offs (online supplemental eTable 11).

Lower HDL <1 mmol/L was associated with a higher risk of dementia in the full cohort. However, with ≥10 years of follow-up, the former association was attenuated. Instead, a higher level of HDL >1.0 mmol/L was associated with a lower risk of dementia. The difference in risks between HDL ≥1.0 mmol/L versus <1.0 mmol/L measured within 10 years of dementia diagnosis was not statistically significant (online supplemental eTable 11). However, the risk of dementia was 12% lower in the group with HDL ≥1.0 mmol/L versus <1.0 mmol/L and over 10 years of follow-up.

### Sensitivity analyses

Findings were robust to sensitivity analyses, including possible dementia cases and adjusting for medication (online supplemental eFigures 2–7 and online supplemental eTables 12 and 13).

With inverse probability weighting, baseline characteristics of individuals were more comparable between subcohorts (online supplemental eTable 1). Splines are presented in online supplemental eFigureA8-A10. Findings were generally robust (online supplemental eTables 14 and 15), but the strength of association for DBP and BMI among those with ≥10 years of follow-up became weaker. For example, HR (95% CI) for the ≥90 mm Hg group was 1.26 (95% CI 1.05 to 1.50) versus without weighting at 1.37 (95% CI 1.24 to 1.51) (online supplemental eTable 14). However, the findings remained largely consistent.

When applying the modified Fine and Gray model in the full cohort, associations remained broadly similar for most study exposures (online supplemental eTables 16 and 17). However, the modified Fine and Gray model attenuated associations for HbA1c and HDL; for example, HbA1c using the Cox model reported 1.07 (95% CI 1.03 to 1.11), but using the modified Fine and Gray gave 1.02 (95% CI 0.98 to 1.06) for >58 mmol/mol versus <48 mmol/mol (online supplemental eTable 16 and 17). When applied to the subcohort with ≥10 years of follow-up, estimates were largely similar to outputs from the Cox regression (online supplemental eTable 18 and 19).

## Discussion

### Summary of findings

To our knowledge, this is the first investigation into the relationship between cardiometabolic measurements and dementia risk in a large cohort of people with T2D that considers the timing of cardiometabolic measurements. The associations observed were complex and non-linear, differing by time to dementia diagnosis. First, SBP was associated with a lower dementia risk, and this association was stronger with shorter follow-up (ie, closer to the diagnosis of dementia), while DBP was mostly associated with a greater risk of dementia. Second, the association between BMI and dementia risk varied by the length of follow-up, whereby within 5 years of dementia diagnosis, higher BMI was associated with a lower dementia risk, but when measured more than 5 years prior to diagnosis, higher BMI was associated with a higher risk of dementia. Third, higher levels of glycemic measures were associated with a greater dementia risk, but this was less pronounced when follow-up was shorter. Fourth, HDL was only linked to lower dementia risk if follow-up was ≥10 years. Lastly, associations reported for total cholesterol and LDL appeared to be short-term, as findings from splines were attenuated with longer follow-up, and analyses using cut-offs largely had no statistically significant differences.

### Blood pressure

The findings for the association between SBP and dementia risk were unexpected. Previous findings from the literature on the T2D population are generally mixed.^5^ Our findings for DBP agreed with some,^6^ but not others.^10^ Trials for T2D-specific populations are lacking and have yet to demonstrate improvements in cognition or dementia risk with intensive blood pressure lowering.^22^ However, observational studies^23^ and trials^24^ within the general population have demonstrated that midlife high blood pressure raises dementia risk^23^ and that intensive blood pressure lowering is beneficial for lowering dementia risk,^24^ though some uncertainty still remains.^25^ As our cohort’s mean age (65 years) does not ideally reflect midlife levels, the levels captured may no longer indicate risk but instead act as markers of disease progression (reverse causation). The weakening inverse association for SBP, inversion of associations for DBP (<75 mm Hg) with longer follow-up time, and the declining trajectories of SBP and DBP over time observed in our previous work all support this explanation.^14^ Hence, DBP might serve as a better long-term indicator of dementia risk than SBP, which might require longer follow-up to reflect risk-relevant values.

### Body mass index

Our findings for BMI and dementia risk likely illustrate the presence of reverse causation. The relationship is non-linear: a higher risk of dementia was only present from 29 kg/m^2^ with ≥10 years of follow-up, while a lower risk of dementia was found with a higher BMI with <5 years of follow-up. Accelerated weight loss occurring from at least a decade before dementia diagnosis in people with T2D was previously reported.^14^ Such divergent associations between BMI and dementia were also observed in general populations^12^ and supported by theories including weight loss, wasting, difficulty in eating, malnutrition, predementia apathy, loss of initiative, leptin reduction, and cognitive dysregulation due to the secretion of hormones and adipokines in the background of age-related weight loss, olfactory dysfunction, and anxiety as potential explanations for this phenomenon.^26^ So far, no trials have demonstrated benefits on cognition with weight loss among people with T2D.^27–29^ Our previous work reported that people with T2D who developed dementia had a consistently lower BMI than those who did not develop dementia throughout 20 years before dementia diagnosis, highlighting the importance of considering the timing of potential interventions.^14^ Therefore, in relation to assessing risk, measurements of BMI should be captured earlier before late life, and non-linear relationships should be considered. Future trials may also need to reconsider the role that BMI plays in dementia development in the context of T2D within their study design.

### Glucose levels

Generally consistent findings for FPG and HbA1c lend support to a possible cumulative effect, given the stronger associations with longer follow-up. Our results align with previous studies^4 5^ and expand upon these findings by including a large T2D population with long follow-up and further stratification based on follow-up time. Established biological mechanisms support this link, whereby prolonged insulin resistance leads to abnormalities in the insulin signaling pathways that affect both systemic metabolism and cerebral insulin signaling.^3^ Current trials, however, have not found sufficient evidence to support the prevention or delay in cognitive dysfunction with intensive glycemic control over standard treatments.^30^ Of note, there is some evidence of non-linearity and reverse causation for the relationship between FPG, HbA1c, and dementia if the follow-up time is less than 5 years, and all the trials included in the systematic review had a follow-up of less than 6 years.^30^ In combination with our findings, it is possible that longer periods of follow-up within trials might be needed to observe benefits with glucose lowering among people with T2D.

### Lipid levels

Overall, total cholesterol and LDL were generally not associated with a greater risk of dementia, and their roles remain inconclusive.^31^ Although higher HDL was associated with a lower risk of dementia with longer follow-up, this association was attenuated with shorter follow-up, and it is unclear whether the latter is due to reverse causation, as HDL levels have been demonstrated to be stable over 20 years prior to dementia diagnosis.^14^ Current proposed biological mechanisms that relate to lipids in general are more indirect and include atherosclerosis, which may in turn influence the development of microvascular and macrovascular disease, established risk factors for dementia.^32^ Existing studies of the T2D population generally reported mixed findings, likely due to the cross-sectional design, short follow-up, small sample sizes, and limited adjustments for confounding.^5^ In trials, intensive statin therapy in combination with fibrates to improve lipid profiles has not proven to be beneficial.^22^ Therefore, the role that cholesterol plays in influencing dementia risk in people with T2D remains to be explored by clinical trials.

### Strengths and limitations

Our study used a large, unselected, and population-based cohort of nearly 250 000 participants with T2D. The retrospective design with two decades of follow-up data allowed exploration by different lengths of follow-up time. CPRD is also broadly representative, with standardized and well-measured key laboratory and clinical parameters, a validated dementia outcome, and quality indicators, which improve accuracy, completeness of the data, and the generalizability of our findings.^15^ Our findings were also robust to a comprehensive sensitivity analysis plan, which included inverse probability weighting to account for any systematic bias in cohort characteristics and competing risk modeling to account for the competing risk of death.

Although CPRD is broadly representative of the population of England in terms of age, sex, and geographical region, the current data may overrepresent patients who are White.^33^ Our analysis assessed baseline levels of cardiometabolic factors in relation to dementia risk and did not account for changes over time, as we intended to assess dementia risk using measurements taken early during the follow-up. Dementia is commonly underdiagnosed, but we enhanced detection through an established algorithm that considered several data sources. We could not assess the level of misclassification caused by coding errors or undiagnosed cases but identified possible dementia cases through our algorithm to identify individuals who may have been lost to follow-up before receiving a clinical diagnosis, including individuals with severe cognitive decline who moved to care homes due to incipient dementia. The subsequent inclusion of these individuals in sensitivity analyses helped to assess the reliability and robustness of our findings, which remained largely consistent. Any residual presence of potential misclassification would likely underestimate group differences. Although we accounted for key confounders, residual confounding and unobserved confounders such as diet and physical activity that are not captured in the CPRD could have impacted the results.

## Conclusion

Higher DBP, BMI, and glucose levels measured over 10 years before dementia diagnosis were linked to a greater dementia risk among people with T2D. However, these measures were not consistently linked to a greater dementia risk when recorded less than 10 years before dementia diagnosis. Associations varied greatly depending on when cardiometabolic factors were measured. Our findings may explain the variability in the findings of previous studies and demonstrate the importance of time-stratified analyses in individual studies and evidence syntheses. Future trials and evidence syntheses should also consider the timeframe of the measurement of cardiometabolic factors to assess their potential role (as a risk factor or marker of disease) within their study design in the context of dementia development in the T2D population.

## Supplementary material

10.1136/bmjdrc-2025-005743 online supplemental file 1

## Data Availability

Data may be obtained from a third party and are not publicly available.
