# Supplementary material for: Cardiometabolic factors and risk of dementia in people with type 2 diabetes in England: a large retrospective cohort study
Source: BMJ Open Diabetes Res Care. 2026 Aug 17;14(4):e005743. doi: 10.1136/bmjdrc-2025-005743 (PMC13504886; doi:10.1136/bmjdrc-2025-005743)
Supplement: Supplementary file 1 [file bmjdrc-14-4-s001.docx]

**Online Supplemental Material**

**Tables**

[Table A.1. Baseline participant characteristics for the full cohort and sub-cohorts by follow-up time with inverse probability weighting in the Clinical Research Practice Datalink in England	4](#_Toc170933885)

[Table A.1. Baseline participant characteristics in the Clinical Research Practice Datalink by systolic blood pressure groups in England	5](#_Toc170933886)

[Table A.2. Baseline participant characteristics in the Clinical Research Practice Datalink by diastolic blood pressure groups in England	6](#_Toc170933887)

[Table A.3. Baseline participant characteristics in the Clinical Research Practice Datalink by body mass index groups in England	7](#_Toc170933888)

[Table A.4. Baseline participant characteristics in the Clinical Research Practice Datalink by fasting plasma glucose groups in England	8](#_Toc170933889)

[Table A.5. Baseline participant characteristics in the Clinical Research Practice Datalink by haemoglobin A1c groups in England	9](#_Toc170933890)

[Table A.6. Baseline participant characteristics in the Clinical Research Practice Datalink by total cholesterol groups in England	10](#_Toc170933891)

[Table A.7. Baseline participant characteristics in the Clinical Research Practice Datalink by high-density lipoprotein groups in England	11](#_Toc170933892)

[Table A.8. Baseline participant characteristics in the Clinical Research Practice Datalink by low-density lipoprotein groups in England	12](#_Toc170933893)

[Table A.9. The association between blood pressure, body mass index, glycaemic measures, and risk of dementia among people with type 2 diabetes in the Clinical Practice Research Datalink in England, evaluated using Cox regression, and stratified by follow-up periods.	13](#_Toc170933894)

[Table A.10. The association between total cholesterol, high-density lipoprotein, and low-density lipoprotein, and risk of dementia among people with type 2 diabetes in the Clinical Practice Research Datalink in England, evaluated using Cox regression, and stratified by follow-up periods.	16](#_Toc170933895)

[Table A.11. The association between blood pressure, body mass index, glycaemic measures, and risk of dementia among people with type 2 diabetes in the Clinical Practice Research Datalink in England, evaluated using Cox regression, (1) including possible dementia cases and (2) adjusting for medications.	24](#_Toc170933896)

[Table A.12. The association between total cholesterol, high-density lipoprotein, low-density lipoprotein, and risk of dementia among people with type 2 diabetes in the Clinical Practice Research Datalink in England, evaluated using Cox regression, (1) including possible dementia cases and (2) adjusting for medications.	26](#_Toc170933897)

[Table A.14. The association between blood pressure, body mass index, glycaemic measures, and risk of dementia among people with type 2 diabetes in the Clinical Practice Research Datalink in England, evaluated using Cox regression, stratified by follow-up periods and weighted using inverse probability weights.	30](#_Toc170933898)

[Table A.15. The association between total cholesterol, high-density lipoprotein, and low-density lipoprotein, and risk of dementia among people with type 2 diabetes in the Clinical Practice Research Datalink in England, evaluated using Cox regression, stratified by follow-up periods and weighted using inverse probability weights.	32](#_Toc170933899)

[Table A.16. The association between blood pressure, body mass index, glycaemic measures, and risk of dementia among people with type 2 diabetes in the Clinical Practice Research Datalink in England, evaluated using Cox regression and a modified Fine and Gray model.	34](#_Toc170933900)

[Table A.17. The association between total cholesterol, high-density lipoprotein, and low-density lipoprotein, and risk of dementia among people with type 2 diabetes in the Clinical Practice Research Datalink in England, evaluated using Cox regression and a modified Fine and Gray model.	35](#_Toc170933901)

[Table A.18. The association between blood pressure, body mass index, glycaemic measures, and risk of dementia among people with type 2 diabetes and more than 10 years of follow-up in the Clinical Practice Research Datalink in England, evaluated using Cox regression and a modified Fine and Gray model.	36](#_Toc170933902)

[Table A.19. The association between total cholesterol, high-density lipoprotein, and low-density lipoprotein, and risk of dementia among people with type 2 diabetes and more than 10 years of follow-up in the Clinical Practice Research Datalink in England, evaluated using Cox regression and a modified Fine and Gray model.	37](#_Toc170933903)

Figures

[Figure A.1. Study flowchart for obtaining the analytical population in the Clinical Practice Research Datalink with linkage to Hospital Episode Statistics (HES) and Office of National Statistics (ONS) mortality data in England.	3](#_Toc170933904)

[Figure A.2. The association between (A) systolic blood pressure, (B) diastolic blood pressure, (C) body mass index, and risk of dementia (including possible dementia cases) among people with type 2 diabetes in the Clinical Practice Research Datalink in England, evaluated using restricted cubic splines, and stratified by follow-up periods.	18](#_Toc170933905)

[Figure A.3. The association between (A) fasting plasma glucose, (B) haemoglobin A1c, and risk of dementia (including possible dementia cases) among people with type 2 diabetes in the Clinical Practice Research Datalink in England, evaluated using restricted cubic splines, and stratified by follow-up periods.	19](#_Toc170933906)

[Figure A.4. The association between (A) total cholesterol, (B) high-density lipoprotein, (C) low-density lipoprotein, and risk of dementia (including possible dementia cases) among people with type 2 diabetes in the Clinical Practice Research Datalink in England, evaluated using restricted cubic splines, and stratified by follow-up periods.	20](#_Toc170933907)

[Figure A.5. The association between (A) systolic blood pressure, (B) diastolic blood pressure, (C) body mass index, and risk of dementia (adjusting for medication use) among people with type 2 diabetes in the Clinical Practice Research Datalink in England, evaluated using restricted cubic splines, and stratified by follow-up periods.	21](#_Toc170933908)

[Figure A.6. The association between (A) fasting plasma glucose, (B) haemoglobin A1c, and risk of dementia (adjusting for medication use) among people with type 2 diabetes in the Clinical Practice Research Datalink in England, evaluated using restricted cubic splines, and stratified by follow-up periods.	22](#_Toc170933909)

[Figure A.7. The association between (A) total cholesterol, (B) high-density lipoprotein, (C) low-density lipoprotein, and risk of dementia (adjusting for medication use) among people with type 2 diabetes in the Clinical Practice Research Datalink in England, evaluated using restricted cubic splines, and stratified by follow-up periods.	23](#_Toc170933910)

[Figure A.8. The association between (A) systolic blood pressure, (B) diastolic blood pressure, (C) body mass index, and risk of dementia among people with type 2 diabetes in the Clinical Practice Research Datalink in England, evaluated using restricted cubic splines, and stratified by follow-up periods (inverse probability weighted).	27](#_Toc170933911)

[Figure A.9. The association between (A) fasting plasma glucose, (B) haemoglobin A1c, and risk of dementia among people with type 2 diabetes in the Clinical Practice Research Datalink in England, evaluated using restricted cubic splines, and stratified by follow-up periods (inverse probability weighted).	28](#_Toc170933912)

[Figure A.10. The association between (A) total cholesterol, (B) high-density lipoprotein, (C) low-density lipoprotein, and risk of dementia among people with type 2 diabetes in the Clinical Practice Research Datalink in England, evaluated using restricted cubic splines, and stratified by follow-up periods (inverse probability weighted).	29](#_Toc170933913)

**Figure A.1. Study flowchart for obtaining the analytical population in the Clinical Practice Research Datalink with linkage to Hospital Episode Statistics (HES) and Office of National Statistics (ONS) mortality data in England.**

Up-to-standard dates are determined by CPRD for general practices, depending on the continuity of patient and death records, which define a period where practices meet the criteria; patient acceptability is a quality metric based on valid age and gender, registration status, and recording of events [2]. All patients in the linked dataset had acceptable data.

**eTable 1. Baseline participant characteristics for the full cohort and sub-cohorts by follow-up time with inverse probability weighting in the Clinical Research Practice Datalink in England**

| **Variables** | **Overall** | **Follow- up time (inverse probability weighted)** | | |
| --- | --- | --- | --- | --- |
|  |  | **<5 years** | **5-10 years** | **≥10 years** |
| **Participants, n** | 249,958 | 143,257 | 71,075 | 35,626 |
| Age, years, mean (SD) | 65.5 (11.7) | 65.3 (12.2) | 65.1 (11.2) | 65.4 (9.9) |
| Sex, n (%) |  |  |  |  |
| Male | 133,596 (53.4) | 77,154 (53.9) | 38,090 (53.6) | 19,357 (54.3) |
| Female | 116,362 (46.6) | 66,103 (46.1) | 32,985 (46.4) | 16,269 (45.7) |
| Ethnicity, n (%) |  |  |  |  |
| White | 210,448 (84.2) | 120,424 (84.1) | 59,863 (84.2) | 30,403 (85.3) |
| Non-White | 26,046 (10.4) | 14,849 (10.4) | 7,280 (10.2) | 2,668 (7.5) |
| Unknown/missing | 13,464 (5.4) | 7,984 (5.6) | 3,932 (5.5) | 2,555 (7.2) |
| Smoking status, n (%) |  |  |  |  |
| Non-smoker | 94,580 (37.8) | 54,010 (37.7) | 26,720 (37.6) | 12,387 (34.8) |
| Smoker | 81,311 (32.5) | 47,025 (32.8) | 22,994 (32.4) | 11,923 (33.5) |
| Ex-smoker | 74,067 (29.6) | 42,221 (29.5) | 21,361 (30.1) | 11,316 (31.8) |
| Index of Multiple Deprivation (IMD) quintiles, n (%) |  |  |  |  |
| 1st quintile (least deprived) | 34,822 (13.9) | 19,526 (13.6) | 9,925 (14.0) | 4,899 (13.8) |
| 2 | 47,271 (18.9) | 27,322 (19.1) | 13,416 (18.9) | 6,206 (17.4) |
| 3 | 48,349 (19.3) | 27,866 (19.5) | 13,671 (19.2) | 6,860 (19.3) |
| 4 | 55,579 (22.2) | 31,491 (22.0) | 15,621 (22.0) | 7,907 (22.2) |
| 5th quintile (most deprived) | 63,937 (25.6) | 37,053 (25.9) | 18,442 (26.0) | 9,754 (27.4) |
| Diabetes duration, years, mean (SD) | 1.84 (3.93) | 1.67 (3.56) | 1.79 (4.62) | 1.40 (3.37) |
| **Medication and comorbidities** |  |  |  |  |
| Anti-hypertensive medication, n (%) | 152,587 (61.0) | 88,306 (61.6) | 44,214 (62.2) | 24,120 (67.7) |
| Anti-lipid medication, n (%) | 128,749 (51.5) | 75,028 (52.4) | 37,273 (52.4) | 20,155 (56.6) |
| Oral anti-hyperglycaemic medication, n (%) | 103,556 (41.4) | 60,793 (42.4) | 30,773 (43.3) | 17,215 (48.3) |
| Insulin, ever prescribed, n (%) | 12,968 (5.2) | 7,554 (5.3) | 4,022 (5.7) | 2,137 (6.0) |
| Anti-platelet medication, n (%) | 64,811 (25.9) | 38,106 (26.6) | 18,900 (26.6) | 11,317 (31.8) |
| Number of co-morbidities, median (IQR)**^*^** | 0 (0-1) | 0 (0-1) | 0 (0-1) | 0 (1-1) |

All listed variables were included in a logistic regression model to generate the inverse probability of being included in the sub-cohort.

^*^Comorbidities include stroke, acute myocardial infarction, peripheral artery disease, atrial fibrillation, heart failure, asthma, chronic obstructive pulmonary disease, cancer, chronic kidney disease, retinopathy, rheumatoid arthritis, Parkinson’s disease, and clinical depression.

**eTable 2. Baseline participant characteristics in the Clinical Research Practice Datalink by systolic blood pressure groups in England**

| **Variables** | **Systolic blood pressure (mmHg)** | | | ***p-value***^*^ |
| --- | --- | --- | --- | --- |
|  | **<130 mmHg** | **130 – 139 mmHg** | **≥140 mmHg** |  |
| **Participants, n** | 63,711 | 64,585 | 109,575 |  |
| Age, years, mean (SD) | 64.0 (12.3) | 65.0 (11.5) | 66.8 (11.2) | <0.001 |
| Sex, n (%) |  |  |  |  |
| Male | 34,781 (54.6) | 35,398 (54.8) | 57,400 (52.4) | <0.001 |
| Female | 28,930 (45.4) | 29,187 (45.2) | 52,175 (47.6) |  |
| Ethnicity, n (%) |  |  |  |  |
| White | 52,493 (82.4) | 54,310 (84.1) | 93,578 (85.4) | <0.001 |
| Non-White | 8,329 (13.1) | 6,953 (10.8) | 9,442 (8.6) |  |
| Unknown/missing | 2,889 (4.5) | 3,322 (5.1) | 6,555 (6.0) |  |
| Smoking status, n (%) |  |  |  |  |
| Non-smoker | 23,711 (37.2) | 24,237 (37.5) | 41,198 (37.6) | <0.001 |
| Smoker | 22,407 (35.2) | 21,030 (32.6) | 34,080 (31.1) |  |
| Ex-smoker | 17,593 (27.6) | 19,318 (29.9) | 34,297 (31.3) |  |
| Index of Multiple Deprivation (IMD) quintiles, n (%) |  |  |  |  |
| 1^st^ quintile (least deprived) | 8,545 (13.4) | 9,078 (14.1) | 14,791 (13.5) | <0.001 |
| 2 | 11,761 (18.5) | 12,169 (18.8) | 21,179 (19.3) |  |
| 3 | 12,368 (19.4) | 12,584 (19.5) | 20,964 (19.1) |  |
| 4 | 14,238 (22.3) | 14,215 (22.0) | 24,841 (22.7) |  |
| 5^th^ quintile (most deprived) | 16,799 (26.4) | 16,539 (25.6) | 27,800 (25.4) |  |
| Diabetes duration, years, mean (SD) | 1.84 (3.93) | 1.64 (3.68) | 1.58 (3.56) | <0.001 |
| Follow-up duration, years, mean (SD) | 4.40 (3.80) | 5.01 (3.95) | 5.58 (4.12) | <0.001 |
| **Medication and comorbidities** |  |  |  |  |
| Insulin, ever prescribed, n (%) | 3,787 (5.9) | 3,139 (4.9) | 5,351 (4.9) | <0.001 |
| Number of co-morbidities, median (IQR)**^†^** | 0 (0-2) | 0 (0-2) | 0 (0-2) | <0.001 |
| Anti-hypertensive medication, n (%) | 32,989 (51.8) | 38,279 (59.3) | 76,997 (70.3) | <0.001 |
| Anti-lipid medication, n (%) | 31,634 (49.7) | 34,824 (53.9) | 58,384 (53.3) | <0.001 |
| Oral anti-hyperglycaemic medication, n (%) | 24,847 (39.0) | 26,992 (41.8) | 47,974 (43.8) | <0.001 |
| Anti-platelet medication, n (%) | 16,800 (26.4) | 17,021 (26.4) | 28,898 (26.4) | 0.996 |

^*^Two-sample independent t-test for continuous variables and chi^2^ test for categorical variables were performed to compare groups stratified by follow-up time.

**^†^**Comorbidities include stroke, acute myocardial infarction, peripheral artery disease, atrial fibrillation, heart failure, asthma, chronic obstructive pulmonary disease, cancer, chronic kidney disease, retinopathy, rheumatoid arthritis, Parkinson’s disease, and clinical depression.

**eTable 3. Baseline participant characteristics in the Clinical Research Practice Datalink by diastolic blood pressure groups in England**

| **Variables** | **Diastolic blood pressure (mmHg)** | | | ***p-value***^*^ |
| --- | --- | --- | --- | --- |
|  | **<80 mmHg** | **80 – 89 mmHg** | **≥90 mmHg** |  |
| **Participants, n** | 117,938 | 90,770 | 29,154 |  |
| Age, years, mean (SD) | 68.6 (11.6) | 63.4 (10.9) | 59.6 (10.3) | <0.001 |
| Sex, n (%) |  |  |  |  |
| Male | 60,661 (51.4) | 49,567 (54.6) | 17,344 (59.5) | <0.001 |
| Female | 57,277 (48.6) | 41,203 (45.4) | 11,810 (40.5) |  |
| Ethnicity, n (%) |  |  |  |  |
| White | 101,372 (86.0) | 75,505 (83.2) | 23,495 (80.6) | <0.001 |
| Non-White | 11,564 (9.8) | 9,752 (10.7) | 3,408 (11.7) |  |
| Unknown/missing | 5,002 (4.2) | 5,513 (6.1) | 2,251 (7.7) |  |
| Smoking status, n (%) |  |  |  |  |
| Non-smoker | 42,841 (36.3) | 34,691 (38.2) | 11,612 (39.8) | <0.001 |
| Smoker | 37,495 (31.8) | 30,065 (33.1) | 9,955 (34.1) |  |
| Ex-smoker | 37,602 (31.9) | 26,014 (28.7) | 7,587 (26.0) |  |
| Index of Multiple Deprivation (IMD) quintiles, n (%) |  |  |  |  |
| 1^st^ quintile (least deprived) | 16,374 (13.9) | 12,265 (13.5) | 3,774 (12.9) | <0.001 |
| 2 | 22,031 (18.7) | 17,278 (19.0) | 5,798 (19.9) |  |
| 3 | 23,162 (19.6) | 17,343 (19.1) | 5,411 (18.6) |  |
| 4 | 26,165 (22.2) | 20,548 (22.6) | 6,577 (22.6) |  |
| 5^th^ quintile (most deprived) | 30,206 (25.6) | 23,336 (25.7) | 7,594 (26.0) |  |
| Diabetes duration, years, mean (SD) | 2.00 (4.24) | 1.40 (3.15) | 1.11 (2.60) | <0.001 |
| Follow-up duration, years, mean (SD) | 4.74 (3.87) | 5.44 (4.11) | 5.60 (4.18) | <0.001 |
| **Medication and comorbidities** |  |  |  |  |
| Insulin, ever prescribed, n (%) | 7,658 (6.5) | 3,668 (4.0) | 951 (3.3) | <0.001 |
| Number of co-morbidities, median (IQR)**^†^** | 1 (0-2) | 1 (0-2) | 1 (0-2) | <0.001 |
| Anti-hypertensive medication, n (%) | 77,923 (66.1) | 53,545 (59.0) | 16,795 (57.6) | <0.001 |
| Anti-lipid medication, n (%) | 68,116 (57.8) | 44,937 (49.5) | 11,785 (40.4) | <0.001 |
| Oral anti-hyperglycaemic medication, n (%) | 51,063 (43.3) | 37,563 (41.4) | 11,184 (38.4) | <0.001 |
| Anti-platelet medication, n (%) | 36,867 (31.3) | 20,620 (22.7) | 5,229 (17.9) | <0.001 |

^*^Two-sample independent t-test for continuous variables and chi^2^ test for categorical variables were performed to compare groups stratified by follow-up time.

**^†^**Comorbidities include stroke, acute myocardial infarction, peripheral artery disease, atrial fibrillation, heart failure, asthma, chronic obstructive pulmonary disease, cancer, chronic kidney disease, retinopathy, rheumatoid arthritis, Parkinson’s disease, and clinical depression.

**eTable 4. Baseline participant characteristics in the Clinical Research Practice Datalink by body mass index groups in England**

| **Variables** | **Body mass index (kg/m^2^)** | | | ***p-value***^*^ |
| --- | --- | --- | --- | --- |
|  | **<25 kg/m^2^** | **25 – 29.9 kg/m^2^** | **≥30 kg/m^2^** |  |
| **Participants, n** | 36,241 | 71,688 | 98,946 |  |
| Age, years, mean (SD) | 69.4 (12.3) | 66.1 (11.0) | 62.1 (10.3) | <0.001 |
| Sex, n (%) |  |  |  |  |
| Male | 18,355 (50.6) | 43,547 (60.7) | 51,342 (51.9) | <0.001 |
| Female | 17,886 (49.4) | 28,141 (39.3) | 47,604 (48.1) |  |
| Ethnicity, n (%) |  |  |  |  |
| White | 29,071 (80.2) | 59,141 (82.5) | 85,902 (86.8) | <0.001 |
| Non-White | 51,54 (14.2) | 8,600 (12.0) | 8,024 (8.1) |  |
| Unknown/missing | 2,016 (5.6) | 3,947 (5.5) | 5,020 (5.1) |  |
| Smoking status, n (%) |  |  |  |  |
| Non-smoker | 14,648 (40.4) | 25,840 (36.0) | 34,578 (34.9) | <0.001 |
| Smoker | 11,587 (32.0) | 22,984 (32.1) | 34,318 (34.7) |  |
| Ex-smoker | 10,006 (27.6) | 22,864 (31.9) | 30,050 (30.4) |  |
| Index of Multiple Deprivation (IMD) quintiles, n (%) |  |  |  |  |
| 1^st^ quintile (least deprived) | 5,494 (15.2) | 10,427 (14.5) | 12,481 (12.6) | <0.001 |
| 2 | 7,013 (19.4) | 13,825 (19.3) | 17,918 (18.1) |  |
| 3 | 6,869 (19.0) | 13,743 (19.2) | 18,827 (19.0) |  |
| 4 | 7,989 (22.0) | 16,236 (22.6) | 22,631 (22.9) |  |
| 5^th^ quintile (most deprived) | 8,876 (24.5) | 17,457 (24.4) | 27,089 (27.4) |  |
| Diabetes duration, years, mean (SD) | 2.08 (4.45) | 1.71 (3.64) | 1.53 (3.33) | <0.001 |
| Follow-up duration, years, mean (SD) | 4.80 (3.97) | 5.52 (4.12) | 5.38 (4.02) | <0.001 |
| **Medication and comorbidities** |  |  |  |  |
| Insulin, ever prescribed, n (%) | 2,549 (7.0) | 3,501 (4.9) | 4,375 (4.4) | <0.001 |
| Number of co-morbidities, median (IQR)**^†^** | 1 (0-2) | 1 (0-2) | 1 (0-2) | <0.001 |
| Anti-hypertensive medication, n (%) | 21,001 (57.9) | 44,519 (62.1) | 61,752 (62.4) | <0.001 |
| Anti-lipid medication, n (%) | 17,632 (48.7) | 40,381 (56.3) | 52,914 (53.5) | <0.001 |
| Oral anti-hyperglycaemic medication, n (%) | 14,507 (40.0) | 31,593 (44.1) | 44,088 (44.6) | <0.001 |
| Anti-platelet medication, n (%) | 9,827 (27.1) | 19,850 (27.7) | 24,155 (24.4) | <0.001 |

^*^Two-sample independent t-test for continuous variables and chi^2^ test for categorical variables were performed to compare groups stratified by follow-up time.

**^†^**Comorbidities include stroke, acute myocardial infarction, peripheral artery disease, atrial fibrillation, heart failure, asthma, chronic obstructive pulmonary disease, cancer, chronic kidney disease, retinopathy, rheumatoid arthritis, Parkinson’s disease, and clinical depression.

**eTable 5. Baseline participant characteristics in the Clinical Research Practice Datalink by fasting plasma glucose groups in England**

| **Variables** | **Fasting plasma glucose** | | | ***p-value***^*^ |
| --- | --- | --- | --- | --- |
|  | **<7.0 mmol/L** | **7.0 – 8.5 mmol/L** | **>8.5 mmol/L** |  |
| **Participants, n** | 63,155 | 49,391 | 70,863 |  |
| Age, years, mean (SD) | 66.7 (11.7) | 65.85 (11.1) | 64.0 (11.5) | <0.001 |
| Sex, n (%) |  |  |  |  |
| Male | 31,138 (49.3) | 27,124 (54.9) | 40,897 (57.7) | <0.001 |
| Female | 32,017 (50.7) | 22,267 (45.1) | 29,966 (42.3) |  |
| Ethnicity, n (%) |  |  |  |  |
| White | 53,527 (84.8) | 42,213 (85.5) | 59,696 (84.2) | <0.001 |
| Non-White | 68,58 (10.9) | 44,62 (9.0) | 6,249 (8.8) |  |
| Unknown/missing | 2,770 (4.4) | 2,716 (5.5) | 4,918 (6.9) |  |
| Smoking status, n (%) |  |  |  |  |
| Non-smoker | 14,648 (40.4) | 25,840 (36.0) | 34,578 (34.9) | <0.001 |
| Smoker | 11,587 (32.0) | 22,984 (32.1) | 34,318 (34.7) |  |
| Ex-smoker | 10,006 (27.6) | 22,864 (31.9) | 30,050 (30.4) |  |
| Index of Multiple Deprivation (IMD) quintiles, n (%) |  |  |  |  |
| 1^st^ quintile (least deprived) | 5,494 (15.2) | 10,427 (14.5) | 12,481 (12.6) | <0.001 |
| 2 | 7,013 (19.4) | 13,825 (19.3) | 17,918 (18.1) |  |
| 3 | 6,869 (19.0) | 13,743 (19.2) | 18,827 (19.0) |  |
| 4 | 7,989 (22.0) | 16,236 (22.6) | 22,631 (22.9) |  |
| 5^th^ quintile (most deprived) | 8,876 (24.5) | 17,457 (24.4) | 27,089 (27.4) |  |
| Diabetes duration, years, mean (SD) | 1.21 (2.99) | 0.97 (2.65) | 1.49 (3.52) | <0.001 |
| Follow-up duration, years, mean (SD) | 5.10 (3.90) | 5.68 (4.00) | 5.78 (4.17) | <0.001 |
| **Medication and comorbidities** |  |  |  |  |
| Insulin, ever prescribed, n (%) | 2,549 (7.0) | 3,501 (4.9) | 4,375 (4.4) | <0.001 |
| Number of co-morbidities, median (IQR) **^†^** | 1 (0-1) | 1 (0-1) | 1 (0-1) | <0.001 |
| Anti-hypertensive medication, n (%) | 21,001 (57.9) | 44,519 (62.1) | 61,752 (62.4) | <0.001 |
| Anti-lipid medication, n (%) | 17,632 (48.7) | 40,381 (56.3) | 52,914 (53.5) | <0.001 |
| Oral anti-hyperglycaemic medication, n (%) | 14.507 (40.0) | 31,593 (44.1) | 44,088 (44.6) | <0.001 |
| Anti-platelet medication, n (%) | 9,827 (27.1) | 19,850 (27.7) | 24,155 (24.4) | <0.001 |

^*^Two-sample independent t-test for continuous variables and chi^2^-squared test for categorical variables were performed to compare groups stratified by follow-up time.

**^†^**Comorbidities include stroke, acute myocardial infarction, peripheral artery disease, atrial fibrillation, heart failure, asthma, chronic obstructive pulmonary disease, cancer, chronic kidney disease, retinopathy, rheumatoid arthritis, Parkinson’s disease, and clinical depression.

**eTable 6. Baseline participant characteristics in the Clinical Research Practice Datalink by haemoglobin A1c groups in England**

| **Variables** | **Haemoglobin A1c** | | | ***p-value***^*^ |
| --- | --- | --- | --- | --- |
|  | **<48 mmol/mol** | **48 – 58 mmol/mol** | **>58 mmol/mol** |  |
| **Participants, n** | 58,890 | 69,380 | 65,818 |  |
| Age, years, mean (SD) | 67.0 (11.4) | 66.3 (11.3) | 62.9 (11.2) | <0.001 |
| Sex, n (%) |  |  |  |  |
| Male | 31,468 (53.4) | 37,117 (53.5) | 38,152 (58.0) | <0.001 |
| Female | 27,422 (46.6) | 32,263 (46.5) | 27,666 (42.0) |  |
| Ethnicity, n (%) |  |  |  |  |
| White | 50,872 (86.4) | 57,792 (83.3) | 53,206 (80.8) | <0.001 |
| Non-White | 5,189 (8.8) | 7,973 (11.5) | 8,279 (12.6) |  |
| Unknown/missing | 2,829 (4.8) | 3,615 (5.2) | 4,333 (6.6) |  |
| Smoking status, n (%) |  |  |  |  |
| Non-smoker | 21,868 (37.1) | 25,802 (37.2) | 24,142 (36.7) | <0.001 |
| Smoker | 17,871 (30.3) | 22,223 (32.0) | 23,472 (35.7) |  |
| Ex-smoker | 19,151 (32.5) | 21,355 (30.8) | 18,204 (27.7) |  |
| Index of Multiple Deprivation (IMD) quintiles, n (%) |  |  |  |  |
| 1^st^ quintile (least deprived) | 8,397 (14.3) | 9,856 (14.2) | 8,816 (13.4) | <0.001 |
| 2 | 11,514 (19.6) | 12,753 (18.4) | 12,005 (18.2) |  |
| 3 | 11,081 (18.8) | 13,718 (19.8) | 13,133 (20.0) |  |
| 4 | 13,186 (22.4) | 15,089 (21.7) | 14,717 (22.4) |  |
| 5^th^ quintile (most deprived) | 14,712 (25.0) | 17,964 (25.9) | 17,147 (26.1) |  |
| Diabetes duration, years, mean (SD) | 1.42 (3.18) | 1.67 (3.56) | 2.27 (4.45) | <0.001 |
| Follow-up duration, years, mean (SD) | 5.08 (4.03) | 5.27 (4.13) | 5.38 (4.16) | <0.001 |
| **Medication and comorbidities** |  |  |  |  |
| Insulin, ever prescribed, n (%) | 1,169 (2.0) | 2,450 (3.5) | 7,235 (11.0) | <0.001 |
| Number of co-morbidities, median (IQR)**^†^** | 0 (0-1) | 0 (0-1) | 0 (0-1) | <0.001 |
| Anti-hypertensive medication, n (%) | 39,167 (66.5) | 45,810 (66.0) | 36,482 (55.4) | <0.001 |
| Anti-lipid medication, n (%) | 32,066 (54.5) | 40,372 (58.2) | 33,392 (50.7) | <0.001 |
| Oral anti-hyperglycaemic medication, n (%) | 17,294 (29.4) | 31,543 (45.5) | 43,176 (65.6) | <0.001 |
| Anti-platelet medication, n (%) | 16,399 (27.8) | 19,374 (27.9) | 15,730 (23.9) | <0.001 |

^*^Two-sample independent t-test for continuous variables and chi^2^ test for categorical variables were performed to compare groups stratified by follow-up time.

**^†^**Comorbidities include stroke, acute myocardial infarction, peripheral artery disease, atrial fibrillation, heart failure, asthma, chronic obstructive pulmonary disease, cancer, chronic kidney disease, retinopathy, rheumatoid arthritis, Parkinson’s disease, and clinical depression.

**eTable 7. Baseline participant characteristics in the Clinical Research Practice Datalink by total cholesterol groups in England**

| **Variables** | **Total cholesterol** | | ***p-value***^*^ |
| --- | --- | --- | --- |
|  | **≤5.0 mmol/L** | **>5.0 mmol/L** |  |
| **Participants, n** | 124,523 | 86,716 |  |
| Age, years, mean (SD) | 66.3 (11.2) | 63.52 (11.3) | <0.001 |
| Sex, n (%) |  |  |  |
| Male | 75,768 (60.8) | 40,001 (46.1) | <0.001 |
| Female | 48,755 (39.2) | 46,715 (53.9) |  |
| Ethnicity, n (%) |  |  |  |
| White | 104,605 (84.0) | 72,725 (83.9) | <0.001 |
| Non-White | 13,964 (11.2) | 8,466 (9.8) |  |
| Unknown/missing | 5,954 (4.8) | 5,525 (6.4) |  |
| Smoking status, n (%) |  |  |  |
| Non-smoker | 43,999 (35.3) | 33,762 (38.9) | <0.001 |
| Smoker | 40,047 (32.2) | 29,491 (34.0) |  |
| Ex-smoker | 40,477 (32.5) | 23,463 (27.1) |  |
| Index of Multiple Deprivation (IMD) quintiles, n (%) |  |  |  |
| 1^st^ quintile (least deprived) | 17,331 (13.9) | 12,313 (14.2) | <0.001 |
| 2 | 23,432 (18.8) | 16,081 (18.5) |  |
| 3 | 23,858 (19.2) | 16,836 (19.4) |  |
| 4 | 27,433 (22.0) | 19,471 (22.5) |  |
| 5^th^ quintile (most deprived) | 32,469 (26.1) | 22,015 (25.4) |  |
| Diabetes duration, years, mean (SD) | 2.00 (4.01) | 1.07 (2.85) | <0.001 |
| Follow-up duration, years, mean (SD) | 5.19 (4.00) | 5.49 (4.11) | <0.001 |
| **Medication and comorbidities** |  |  |  |
| Insulin, ever prescribed, n (%) | 7,214 (5.8) | 3,114 (3.6) | <0.001 |
| Number of co-morbidities, median (IQR)**^†^** | 1 (0-1) | 0 (0-1) | <0.001 |
| Anti-hypertensive medication, n (%) | 85,672 (68.8) | 46,780 (53.9) | <0.001 |
| Anti-lipid medication, n (%) | 78,011 (62.6) | 38,770 (44.7) | <0.001 |
| Oral anti-hyperglycaemic medication, n (%) | 58,564 (47.0) | 33,574 (38.7) | <0.001 |
| Anti-platelet medication, n (%) | 40,513 (32.5) | 16,332 (18.8) | <0.001 |

^*^Two-sample independent t-test for continuous variables and chi^2^ test for categorical variables were performed to compare groups stratified by follow-up time.

**^†^**Comorbidities include stroke, acute myocardial infarction, peripheral artery disease, atrial fibrillation, heart failure, asthma, chronic obstructive pulmonary disease, cancer, chronic kidney disease, retinopathy, rheumatoid arthritis, Parkinson’s disease, and clinical depression.

**eTable 8. Baseline participant characteristics in the Clinical Research Practice Datalink by high-density lipoprotein groups in England**

| **Variables** | **High-density lipoprotein** | | ***p-value***^*^ |
| --- | --- | --- | --- |
|  | **<1.0 mmol/L** | **≥1.0 mmol/L** |  |
| **Participants, n** | 46,708 | 133,734 |  |
| Age, years, mean (SD) | 62.7 (11.0) | 65.6 (11.2) | <0.001 |
| Sex, n (%) |  |  |  |
| Male | 34,584 (74.0) | 64,469 (48.2) | <0.001 |
| Female | 12,124 (26.0) | 69,265 (51.8) |  |
| Ethnicity, n (%) |  |  |  |
| White | 39,246 (84.0) | 111,469 (83.4) | 0.001 |
| Non-White | 5,023 (10.8) | 15,259 (11.4) |  |
| Unknown/missing | 2,439 (5.2) | 7,006 (5.2) |  |
| Smoking status, n (%) |  |  |  |
| Non-smoker | 14,174 (30.3) | 52,431 (39.2) | <0.001 |
| Smoker | 18,846 (40.3) | 40,906 (30.6) |  |
| Ex-smoker | 13,688 (29.3) | 40,397 (30.2) |  |
| Index of Multiple Deprivation (IMD) quintiles, n (%) |  |  |  |
| 1^st^ quintile (least deprived) | 6,312 (13.5) | 20,496 (15.3) | <0.001 |
| 2 | 8,776 (18.8) | 25,658 (19.2) |  |
| 3 | 8,945 (19.2) | 24,107 (18.0) |  |
| 4 | 10,013 (21.4) | 29,643 (22.2) |  |
| 5^th^ quintile (most deprived) | 12,662 (27.1) | 33,830 (25.3) |  |
| Diabetes duration, years, mean (SD) | 1.61 (3.55) | 1.52 (3.54) | <0.001 |
| Follow-up duration, years, mean (SD) | 5.08 (3.95) | 5.28 (4.05) | <0.001 |
| **Medication and comorbidities** |  |  |  |
| Insulin, ever prescribed, n (%) | 2,279 (4.9) | 5,498 (4.1) | <0.001 |
| Number of co-morbidities, median (IQR)**^†^** | 0 (0-1) | 0 (0-1) | 0.001 |
| Anti-hypertensive medication, n (%) | 28,104 (60.2) | 83,635 (62.5) | <0.001 |
| Anti-lipid medication, n (%) | 25,432 (54.4) | 75,255 (56.3) | <0.001 |
| Oral anti-hyperglycaemic medication, n (%) | 21,683 (46.4) | 55,578 (41.6) | <0.001 |
| Anti-platelet medication, n (%) | 34,440 (25.8) | 34,440 (25.8) | <0.001 |

^*^Two-sample independent t-test for continuous variables and chi^2^ test for categorical variables were performed to compare groups stratified by follow-up time.

**^†^**Comorbidities include stroke, acute myocardial infarction, peripheral artery disease, atrial fibrillation, heart failure, asthma, chronic obstructive pulmonary disease, cancer, chronic kidney disease, retinopathy, rheumatoid arthritis, Parkinson’s disease, and clinical depression.

**eTable 9. Baseline participant characteristics in the Clinical Research Practice Datalink by low-density lipoprotein groups in England**

| **Variables** | **Low-density lipoprotein** | | ***p-value***^*^ |
| --- | --- | --- | --- |
|  | **≤3.0 mmol/L** | **>3.0 mmol/L** |  |
| **Participants, n** | 89,917 | 57,651 |  |
| Age, years, mean (SD) | 66.0 (11.0) | 62.9 (11.0) | <0.001 |
| Sex, n (%) |  |  |  |
| Male | 52,510 (58.4) | 28,237 (49.0) | <0.001 |
| Female | 37,407 (41.6) | 29,414 (51.0) |  |
| Ethnicity, n (%) |  |  |  |
| White | 74,905 (83.3) | 46,855 (81.3) | <0.001 |
| Non-White | 11,016 (12.3) | 7,028 (12.2) |  |
| Unknown/missing | 3,996 (4.4) | 3,768 (6.5) |  |
| Smoking status, n (%) |  |  |  |
| Non-smoker | 32,243 (35.9) | 23,196 (40.2) | <0.001 |
| Smoker | 29,249 (32.5) | 19,175 (33.3) |  |
| Ex-smoker | 28,425 (31.6) | 15,280 (26.5) |  |
| Index of Multiple Deprivation (IMD) quintiles, n (%) |  |  |  |
| 1st quintile (least deprived) | 13,339 (14.8) | 8,496 (14.7) | <0.001 |
| 2 | 16,655 (18.5) | 10,984 (19.1) |  |
| 3 | 15,949 (17.7) | 10,833 (18.8) |  |
| 4 | 20,996 (23.4) | 13,095 (22.7) |  |
| 5^th^ quintile (most deprived) | 22,978 (25.6) | 14,243 (24.7) |  |
| Diabetes duration, years, mean (SD) | 1.87 (3.93) | 0.96 (2.72) | <0.001 |
| Follow-up duration, years, mean (SD) | 5.17 (3.94) | 5.14 (4.02) | 0.091 |
| **Medication and comorbidities** |  |  |  |
| Insulin, ever prescribed, n (%) | 4,436 (4.9) | 1,473 (2.6) | <0.001 |
| Number of co-morbidities, median (IQR)**^†^** | 0 (0-1) | 0 (0-1) | <0.001 |
| Anti-hypertensive medication, n (%) | 61,544 (68.4) | 29,068 (50.4) | <0.001 |
| Anti-lipid medication, n (%) | 57,998 (64.5) | 24,332 (42.2) | <0.001 |
| Oral anti-hyperglycaemic medication, n (%) | 41,268 (45.9) | 20,941 (36.3) | <0.001 |
| Anti-platelet medication, n (%) | 28,565 (31.8) | 9,886 (17.1) | <0.001 |

^*^Two-sample independent t-test for continuous variables and chi^2^ test for categorical variables were performed to compare groups stratified by follow-up time.

**^†^**Comorbidities include stroke, acute myocardial infarction, peripheral artery disease, atrial fibrillation, heart failure, asthma, chronic obstructive pulmonary disease, cancer, chronic kidney disease, retinopathy, rheumatoid arthritis, Parkinson’s disease, and clinical depression.

**eTable 10. The association between blood pressure, body mass index, glycaemic measures, and risk of dementia among people with type 2 diabetes in the Clinical Practice Research Datalink in England, evaluated using Cox regression, and stratified by follow-up periods.**

| **Systolic blood pressure** | **<130 mmHg** | **130 – 139 mmHg** | **≥140 mmHg** |
| --- | --- | --- | --- |
| Events/Total (per 1,000 person-years) | 5,338/63,711 (19) | 5,277/64,585 (16) | 10,914/109,575 (18) |
| Median (IQR), mmHg | 122 (116-126) | 135 (132-137) | 149 (144-157) |
| Sex-adjusted, HR (95% CI) | 1.00 (Ref) | 0.74 (0.72-0.77) | 0.65 (0.62-0.67) |
| Intermediate model, HR (95% CI) | 1.00 (Ref) | 0.79 (0.76-0.82) | 0.73 (0.71-0.76) |
| Fully adjusted, HR (95% CI) | 1.00 (Ref) | 0.80 (0.77-0.83) | 0.74 (0.72-0.77) |
| ***Follow-up <5 years*** |  |  |  |
| Events/Total (per 1,000 person-years) | 3,192/40,865 (39) | 2,480/37,200 (31) | 4,269/56,338 (34) |
| Median (IQR), mmHg | 121 (115-126) | 135 (132-137) | 148 (143-156) |
| Sex-adjusted, HR (95% CI) | 1.00 (Ref) | 0.75 (0.71-0.79) | 0.69 (0.66-0.72) |
| Intermediate model, HR (95% CI) | 1.00 (Ref) | 0.76 (0.72-0.81) | 0.70 (0.67-0.73) |
| Fully adjusted, HR (95% CI) | 1.00 (Ref) | 0.78 (0.74-0.82) | 0.71 (0.68-0.75) |
| ***Follow-up 5-10 years*** |  |  |  |
| Events/Total (per 1,000 person-years) | 1,404/16,079 (12) | 1,715/18,522 (13) | 3,793/34,035 (15) |
| Median (IQR), mmHg | 122 (117-126) | 135 (132-137) | 149 (144-157) |
| Sex-adjusted, HR (95% CI) | 1.00 (Ref) | 0.88 (0.82-0.94) | 0.82 (0.77-0.87) |
| Intermediate model, HR (95% CI) | 1.00 (Ref) | 0.89 (0.83-0.96) | 0.81 (0.76-0.86) |
| Fully adjusted, HR (95% CI) | 1.00 (Ref) | 0.90 (0.83-0.96) | 0.82 (0.77-0.87) |
| ***Follow-up* ≥*10 years*** |  |  |  |
| Events/Total (per 1,000 person-years) | 742/6,767 (9) | 1,082/8,863 (10) | 2,852/19,202 (12) |
| Median (IQR), mmHg | 122 (118-127) | 135 (132-137) | 150 (144-158) |
| Sex-adjusted, HR (95% CI) | 1.00 (Ref) | 0.84 (0.77-0.92) | 0.78 (0.72-0.85) |
| Intermediate model, HR (95% CI) | 1.00 (Ref) | 0.85 (0.78-0.93) | 0.79 (0.73-0.86) |
| Fully adjusted, HR (95% CI) | 1.00 (Ref) | 0.86 (0.78-0.94) | 0.80 (0.74-0.87) |
| **Diastolic blood pressure** | **<80 mmHg** | **80 – 89 mmHg** | **≥90 mmHg** |
| Events/Total (per 1,000 person-years) | 12,974/117,938 (23) | 6,854/90,770 (14) | 1,698/29,154 (10) |
| Median (IQR), mmHg | 73 (70-77) | 83 (81-86) | 93 (91-97) |
| Sex-adjusted, HR (95% CI) | 1.00 (Ref) | 0.94 (0.92-0.97) | 1.06 (1.00-1.11) |
| Intermediate model, HR (95% CI) | 1.00 (Ref) | 1.02 (0.99-1.05) | 1.17 (1.12-1.24) |
| Fully adjusted, HR (95% CI) | 1.00 (Ref) | 1.04 (1.01-1.07) | 1.21 (1.15-1.27) |
| ***Follow-up <5 years*** |  |  |  |
| Events/Total (per 1,000 person-years) | 6,677/71,297 (44) | 2,646/48,186 (25) | 617/14,917 (19) |
| Median (IQR), mmHg | 73 (69-76) | 83 (81-86) | 93 (91-97) |
| Sex-adjusted, HR (95% CI) | 1.00 (Ref) | 0.93 (0.89-0.97) | 1.05 (0.96-1.14) |
| Intermediate model, HR (95% CI) | 1.00 (Ref) | 0.93 (0.89-0.98) | 1.05 (0.96-1.14) |
| Fully adjusted, HR (95% CI) | 1.00 (Ref) | 0.96 (0.91-1.00) | 1.08 (0.99-1.18) |
| ***Follow-up 5-10 years*** |  |  |  |
| Events/Total (per 1,000 person-years) | 3,968/32,285 (17) | 2,347/27,420 (12) | 595/8,929 (9) |
| Median (IQR), mmHg | 74 (70-77) | 83 (81-86) | 93 (91-97) |
| Sex-adjusted, HR (95% CI) | 1.00 (Ref) | 1.11 (1.06-1.17) | 1.33 (1.22-1.45) |
| Intermediate model, HR (95% CI) | 1.00 (Ref) | 1.11 (1.06-1.17) | 1.32 (1.21-1.44) |
| Fully adjusted, HR (95% CI) | 1.00 (Ref) | 1.12 (1.07-1.18) | 1.34 (1.23-1.46) |
| ***Follow-up ≥10 years*** |  |  |  |
| Events/Total (per 1,000 person-years) | 2,329/14,356 (13) | 1,861/15,164 (10) | 486/5,308 (7) |
| Median (IQR), mmHg | 74 (70-77) | 84 (81-86) | 93 (91-97) |
| Sex-adjusted, HR (95% CI) | 1.00 (Ref) | 1.16 (1.09-1.23) | 1.33 (1.20-1.47) |
| Intermediate model, HR (95% CI) | 1.00 (Ref) | 1.17 (1.10-1.24) | 1.34 (1.21-1.48) |
| Fully adjusted, HR (95% CI) | 1.00 (Ref) | 1.19 (1.12-1.27) | 1.37 (1.24-1.51) |
| **Body mass index** | **<25 kg/m^2^** | **25 – 29.9 kg/m^2^** | **≥30 kg/m^2^** |
| Events/Total (per 1,000 person-years) | 4,861/36,241 (28) | 6,578/71,688 (17) | 6,045/98,946 (11) |
| Median (IQR), kg/m^2^ | 23 (21-24) | 28 (26-29) | 34 (32-38) |
| Sex-adjusted, HR (95% CI) | 1.00 (Ref) | 0.81 (0.78-0.84) | 0.88 (0.85-0.92) |
| Intermediate model, HR (95% CI) | 1.00 (Ref) | 0.83 (0.80-0.87) | 0.89 (0.86-0.93) |
| Fully adjusted, HR (95% CI) | 1.00 (Ref) | 0.84 (0.81-0.87) | 0.90 (0.87-0.94) |
| ***Follow-up <5 years*** |  |  |  |
| Events/Total (per 1,000 person-years) | 2,622/21,855 (58) | 2,490/37,442 (30) | 2,021/52,974 (17) |
| Median (IQR), kg/m^2^ | 23 (21-24) | 28 (26-29) | 34 (32-38) |
| Sex-adjusted, HR (95% CI) | 1.00 (Ref) | 0.76 (0.72-0.81) | 0.67 (0.63-0.71) |
| Intermediate model, HR (95% CI) | 1.00 (Ref) | 0.77 (0.73-0.81) | 0.68 (0.64-0.72) |
| Fully adjusted, HR (95% CI) | 1.00 (Ref) | 0.78 (0.74-0.82) | 0.68 (0.64-0.73) |
| ***Follow-up 5-10 years*** |  |  |  |
| Events/Total (per 1,000 person-years) | 1,418/9,635 (20) | 2,332/21,965 (15) | 2,262/30,333 (10) |
| Median (IQR), kg/m^2^ | 23 (22-24) | 28 (26-29) | 34 (32-38) |
| Sex-adjusted, HR (95% CI) | 1.00 (Ref) | 0.97 (0.91-1.04) | 1.12 (1.05-1.20) |
| Intermediate model, HR (95% CI) | 1.00 (Ref) | 0.98 (0.92-1.05) | 1.15 (1.07-1.23) |
| Fully adjusted, HR (95% CI) | 1.00 (Ref) | 0.99 (0.92-1.06) | 1.15 (1.07-1.23) |
| ***Follow-up ≥10 years*** |  |  |  |
| Events/Total (per 1,000 person-years) | 821/4,751 (14) | 1,756/12,281 (11) | 1,762/15,639 (9) |
| Median (IQR), kg/m^2^ | 23 (22-24) | 28 (26-29) | 34 (32-37) |
| Sex-adjusted, HR (95% CI) | 1.00 (Ref) | 1.05 (0.97-1.14) | 1.40 (1.29-1.52) |
| Intermediate model, HR (95% CI) | 1.00 (Ref) | 1.06 (0.98-1.16) | 1.42 (1.31-1.55) |
| Fully adjusted, HR (95% CI) | 1.00 (Ref) | 1.07 (0.98-1.16) | 1.43 (1.31-1.56) |
| **Fasting plasma glucose** | **<7.0 mmol/L** | **7.0 – 8.5 mmol/L** | **>8.5 mmol/L** |
| Events/Total (per 1,000 person-years) | 6,214/63,155 (19) | 4,487/49,391 (16) | 6,339/70,863 (15) |
| Median (IQR), mmol/L | 6 (5-7) | 8 (7-8) | 11 (10-14) |
| Sex-adjusted, HR (95% CI) | 1.00 (Ref) | 0.92 (0.89-0.96) | 1.06 (1.02-1.10) |
| Intermediate model, HR (95% CI) | 1.00 (Ref) | 0.96 (0.93-1.00) | 1.15 (1.11-1.20) |
| Fully adjusted, HR (95% CI) | 1.00 (Ref) | 0.97 (0.93-1.01) | 1.13 (1.09-1.17) |
| ***Follow-up <5 years*** |  |  |  |
| Events/Total (per 1,000 person-years) | 2,949/35,630 (37) | 1,738/24,620 (30) | 2,578/34,923 (33) |
| Median (IQR), mmol/L | 6 (5-6) | 8 (7-8) | 12 (10-15) |
| Sex-adjusted, HR (95% CI) | 1.00 (Ref) | 0.91 (0.86-0.97) | 1.10 (1.04-1.16) |
| Intermediate model, HR (95% CI) | 1.00 (Ref) | 0.92 (0.87-0.98) | 1.10 (1.04-1.16) |
| Fully adjusted, HR (95% CI) | 1.00 (Ref) | 0.93 (0.88-0.99) | 1.06 (1.01-1.12) |
| ***Follow-up 5-10 years*** |  |  |  |
| Events/Total (per 1,000 person-years) | 2,063/18,931 (15) | 1,613/16,439 (14) | 2,074/22,510 (13) |
| Median (IQR), mmol/L | 6 (5-7) | 8 (7-8) | 11 (10-14) |
| Sex-adjusted, HR (95% CI) | 1.00 (Ref) | 0.97 (0.91-1.04) | 1.11 (1.04-1.18) |
| Intermediate model, HR (95% CI) | 1.00 (Ref) | 0.98 (0.91-1.04) | 1.10 (1.03-1.17) |
| Fully adjusted, HR (95% CI) | 1.00 (Ref) | 0.98 (0.92-1.05) | 1.08 (1.02-1.15) |
| ***Follow-up ≥10 years*** |  |  |  |
| Events/Total (per 1,000 person-years) | 1,202/8,594 (11) | 1,136/8,332 (11) | 1,687/13,430 (10) |
| Median (IQR), mmol/L | 6 (6-7) | 8 (7-8) | 11 (10-14) |
| Sex-adjusted, HR (95% CI) | 1.00 (Ref) | 1.10 (1.01-1.19) | 1.24 (1.16-1.34) |
| Intermediate model, HR (95% CI) | 1.00 (Ref) | 1.11 (1.02-1.20) | 1.25 (1.16-1.34) |
| Fully adjusted, HR (95% CI) | 1.00 (Ref) | 1.11 (1.02-1.20) | 1.24 (1.15-1.33) |
| **Haemoglobin A1c** | **<48 mmol/mol** | **48 – 58 mmol/mol** | **>58 mmol/mol** |
| Events/Total (per 1,000 person-years) | 5,890/58,890 (20) | 6,353/69,380 (17) | 5,078/65,818 (14) |
| Median (IQR), mmol/mol | 43 (40-45) | 52 (50-55) | 72 (64-85) |
| Sex-adjusted, HR (95% CI) | 1.00 (Ref) | 0.94 (0.91-0.98) | 1.10 (1.06-1.14) |
| Intermediate model, HR (95% CI) | 1.00 (Ref) | 0.96 (0.92-0.99) | 1.14 (1.09-1.18) |
| Fully adjusted, HR (95% CI) | 1.00 (Ref) | 0.94 (0.91-0.98) | 1.07 (1.03-1.11) |
| ***Follow-up <5 years*** |  |  |  |
| Events/Total (per 1,000 person-years) | 2,803/33,748 (39) | 2,652/38,377 (32) | 2,110/35,634 (27) |
| Median (IQR), mmol/mol | 43 (40-45) | 52 (50-55) | 73 (64-86) |
| Sex-adjusted, HR (95% CI) | 1.00 (Ref) | 0.90 (0.85-0.95) | 1.03 (0.97-1.09) |
| Intermediate model, HR (95% CI) | 1.00 (Ref) | 0.90 (0.85-0.94) | 1.02 (0.96-1.08) |
| Fully adjusted, HR (95% CI) | 1.00 (Ref) | 0.88 (0.84-0.93) | 0.94 (0.89-1.00) |
| ***Follow-up 5-10 years*** |  |  |  |
| Events/Total (per 1,000 person-years) | 1,876/16,499 (16) | 2,019/19,744 (14) | 1,660/19,028 (12) |
| Median (IQR), mmol/mol | 42 (40-45) | 52 (50-55) | 72 (64-84) |
| Sex-adjusted, HR (95% CI) | 1.00 (Ref) | 0.99 (0.93-1.05) | 1.18 (1.11-1.26) |
| Intermediate model, HR (95% CI) | 1.00 (Ref) | 0.98 (0.92-1.04) | 1.16 (1.09-1.24) |
| Fully adjusted, HR (95% CI) | 1.00 (Ref) | 0.97 (0.91-1.03) | 1.13 (1.05-1.21) |
| ***Follow-up ≥10 years*** |  |  |  |
| Events/Total (per 1,000 person-years) | 1,211/8,643 (11) | 1,682/11,259 (12) | 1,308/11,156 (9) |
| Median (IQR), mmol/mol | 42 (38-44) | 53 (50-55) | 69 (64-81) |
| Sex-adjusted, HR (95% CI) | 1.00 (Ref) | 1.09 (1.01-1.18) | 1.31 (1.21-1.42) |
| Intermediate model, HR (95% CI) | 1.00 (Ref) | 1.08 (1.01-1.17) | 1.29 (1.19-1.40) |
| Fully adjusted, HR (95% CI) | 1.00 (Ref) | 1.07 (1.00-1.16) | 1.25 (1.16-1.36) |

Age was used as the timescale for Cox regression. The intermediate model adjusted for sex, ethnicity, Multiple Index of Deprivation, smoking status, and total comorbidities. The fully adjusted model also additionally included diabetes duration and insulin use. CI, confidence interval; HR, hazard ratio; IQR, interquartile range.

**eTable 11. The association between total cholesterol, high-density lipoprotein, and low-density lipoprotein, and risk of dementia among people with type 2 diabetes in the Clinical Practice Research Datalink in England, evaluated using Cox regression, and stratified by follow-up periods.**

| **Total cholesterol** | **≤5.0 mmol/L** | **>5.0 mmol/L** |
| --- | --- | --- |
| Events/Total (per 1,000 person-years) | 11,578/124,523 (18) | 6,889/86,716 (14) |
| Median (IQR), mmol/L | 4 (4-5) | 6 (5-6) |
| Sex-adjusted, HR (95% CI) | 1.00 (Ref) | 0.91 (0.89-0.94) |
| Intermediate model, HR (95% CI) | 1.00 (Ref) | 0.98 (0.95-1.01) |
| Fully-adjusted, HR (95% CI) | 1.00 (Ref) | 1.01 (0.97-1.04) |
| ***Follow-up <5 years*** |  |  |
| Events/Total (per 1,000 person-years) | 5,230/69,460 (34) | 2,658/45,702 (26) |
| Median (IQR), mmol/L | 4 (4-5) | 6 (5-6) |
| Sex-adjusted, HR (95% CI) | 1.00 (Ref) | 0.95 (0.90-0.99) |
| Intermediate model, HR (95% CI) | 1.00 (Ref) | 0.96 (0.91-1.00) |
| Fully-adjusted, HR (95% CI) | 1.00 (Ref) | 0.99 (0.94-1.04) |
| ***Follow-up 5-10 years*** |  |  |
| Events/Total (per 1,000 person-years) | 3,747/36,515 (14) | 2,431/26,384 (13) |
| Median (IQR), mmol/L | 4 (4-5) | 6 (5-6) |
| Sex-adjusted, HR (95% CI) | 1.00 (Ref) | 1.01 (0.95-1.06) |
| Intermediate model, HR (95% CI) | 1.00 (Ref) | 1.01 (0.95-1.06) |
| Fully-adjusted, HR (95% CI) | 1.00 (Ref) | 1.02 (0.96-1.07) |
| ***Follow-up ≥10 years*** |  |  |
| Events/Total (per 1,000 person-years) | 2,601/18,548 (11) | 1,800/14,630 (10) |
| Median (IQR), mmol/L | 4 (4-5) | 6 (5-6) |
| Sex-adjusted, HR (95% CI) | 1.00 (Ref) | 0.94 (0.89-1.00) |
| Intermediate model, HR (95% CI) | 1.00 (Ref) | 0.95 (0.89-1.01) |
| Fully-adjusted, HR (95% CI) | 1.00 (Ref) | 0.96 (0.90-1.02) |
| **High-density lipoprotein** | **<1.0 mmol/L** | **≥1.0 mmol/L** |
| Events/Total (per 1,000 person-years) | 3,034/46,708 (13) | 11,788/133,734 (17) |
| Median (IQR), mmol/L | 1 (1-1) | 1 (1-2) |
| Sex-adjusted, HR (95% CI) | 1.00 (Ref) | 0.90 (0.86-0.93) |
| Intermediate model, HR (95% CI) | 1.00 (Ref) | 0.93 (0.89-0.96) |
| Fully-adjusted, HR (95% CI) | 1.00 (Ref) | 0.93 (0.90-0.97) |
| ***Follow-up <5 years*** |  |  |
| Events/Total (per 1,000 person-years) | 1,370/26,445 (24) | 4,982/73,600 (31) |
| Median (IQR), mmol/L | 1 (1-1) | 1 (1-2) |
| Sex-adjusted, HR (95% CI) | 1.00 (Ref) | 0.93 (0.87-0.99) |
| Intermediate model, HR (95% CI) | 1.00 (Ref) | 0.95 (0.89-1.01) |
| Fully-adjusted, HR (95% CI) | 1.00 (Ref) | 0.96 (0.90-1.02) |
| ***Follow-up 5-10 years*** |  |  |
| Events/Total (per 1,000 person-years) | 1,004/13,904 (10) | 4,031/39,237 (14) |
| Median (IQR), mmol/L | 1 (1-1) | 1 (1-2) |
| Sex-adjusted, HR (95% CI) | 1.00 (Ref) | 0.95 (0.89-1.02) |
| Intermediate model, HR (95% CI) | 1.00 (Ref) | 0.96 (0.89-1.03) |
| Fully-adjusted, HR (95% CI) | 1.00 (Ref) | 0.96 (0.90-1.04) |
| ***Follow-up ≥10 years*** |  |  |
| Events/Total (per 1,000 person-years) | 660/6,359 (8) | 2,775/20,897 (11) |
| Median (IQR), mmol/L | 1 (1-1) | 1 (1-2) |
| Sex-adjusted, HR (95% CI) | 1.00 (Ref) | 0.86 (0.78-0.93) |
| Intermediate model, HR (95% CI) | 1.00 (Ref) | 0.87 (0.80-0.95) |
| Fully-adjusted, HR (95% CI) | 1.00 (Ref) | 0.88 (0.80-0.96) |
| **Low-density lipoprotein** | **≤3.0 mmol/L** | **>3.0 mmol/L** |
| Events/Total (per 1,000 person-years) | 7,823/89,917 (17) | 3,954/57,651 (13) |
| Median (IQR), mmol/L | 2 (2-3) | 4 (3-4) |
| Sex-adjusted, HR (95% CI) | 1.00 (Ref) | 0.93 (0.89-0.96) |
| Intermediate model, HR (95% CI) | 1.00 (Ref) | 0.99 (0.95-1.03) |
| Fully-adjusted, HR (95% CI) | 1.00 (Ref) | 1.02 (0.98-1.06) |
| ***Follow-up <5 years*** |  |  |
| Events/Total (per 1,000 person-years) | 3,481/49,883 (31) | 1,522/32,417 (22) |
| Median (IQR), mmol/L | 2 (2-3) | 4 (3-4) |
| Sex-adjusted, HR (95% CI) | 1.00 (Ref) | 0.94 (0.88-0.99) |
| Intermediate model, HR (95% CI) | 1.00 (Ref) | 0.95 (0.90-1.01) |
| Fully-adjusted, HR (95% CI) | 1.00 (Ref) | 0.99 (0.93-1.05) |
| ***Follow-up 5-10 years*** |  |  |
| Events/Total (per 1,000 person-years) | 2,611/27,177 (13) | 1,426/16,834 (12) |
| Median (IQR), mmol/L | 2 (2-3) | 4 (3-4) |
| Sex-adjusted, HR (95% CI) | 1.00 (Ref) | 1.03 (0.97-1.10) |
| Intermediate model, HR (95% CI) | 1.00 (Ref) | 1.04 (0.97-1.11) |
| Fully-adjusted, HR (95% CI) | 1.00 (Ref) | 1.05 (0.98-1.12) |
| ***Follow-up ≥10 years*** |  |  |
| Events/Total (per 1,000 person-years) | 1,731/12,857 (11) | 1,006/8,400 (10) |
| Median (IQR), mmol/L | 2 (2-3) | 4 (3-4) |
| Sex-adjusted, HR (95% CI) | 1.00 (Ref) | 0.93 (0.86-1.01) |
| Intermediate model, HR (95% CI) | 1.00 (Ref) | 0.94 (0.87-1.02) |
| Fully-adjusted, HR (95% CI) | 1.00 (Ref) | 0.95 (0.88-1.03) |

Age was used as the timescale for Cox regression. The intermediate model adjusted for sex, ethnicity, Multiple Index of Deprivation, smoking status, and total comorbidities. The fully adjusted model also additionally included diabetes duration and insulin use. CI, confidence interval; HR, hazard ratio; IQR, interquartile range.

**eFigure 2. The association between (A) systolic blood pressure, (B) diastolic blood pressure, (C) body mass index, and risk of dementia (including possible dementia cases) among people with type 2 diabetes in the Clinical Practice Research Datalink in England, evaluated using restricted cubic splines, and stratified by follow-up periods.**

The solid lines and shaded area represent the central risk estimate and 95% CI confidence interval, respectively. The dotted vertical lines are reference lines, corresponding to (A) 120, 130, 140, 150, 160 mmHg, (B) 70, 75, 80, 85, 90 mmHg, and (C) 20, 25, 30, 35, 40 kg/m2. The top and bottom 1% of participants were omitted as outliers to provide better visualisation. Evidence for non-linearity (P_curve_) was calculated by performing a likelihood ratio test between a multivariable model with all spline terms vs. a multivariable model with only the linear term. Model adjustments include sex, ethnicity, calendar year of entry, Index of Multiple Deprivation, smoking, total comorbidities, insulin use, and diabetes duration.

**eFigure 3. The association between (A) fasting plasma glucose, (B) haemoglobin A1c, and risk of dementia (including possible dementia cases) among people with type 2 diabetes in the Clinical Practice Research Datalink in England, evaluated using restricted cubic splines, and stratified by follow-up periods.**

The solid lines and shaded area represent the central risk estimate and 95% CI confidence interval, respectively. The dotted vertical lines are reference lines, corresponding to (A) 5, 6, 7, 8, 9 mmol/L and (B) 43, 48, 53, 58 mmol/mol. The top and bottom 1% of participants were omitted as outliers to provide better visualisation. Evidence for non-linearity (P_curve_) was calculated by performing a likelihood ratio test between a multivariable model with all spline terms vs. a multivariable model with only the linear term. Model adjustments include sex, ethnicity, calendar year of entry, Index of Multiple Deprivation, smoking, total comorbidities, insulin use, and diabetes duration.

**eFigure 4****. The association between (A) total cholesterol, (B) high-density lipoprotein, (C) low-density lipoprotein, and risk of dementia (including possible dementia cases) among people with type 2 diabetes in the Clinical Practice Research Datalink in England, evaluated using restricted cubic splines, and stratified by follow-up periods.**

The solid lines and shaded area represent the central risk estimate and 95% CI confidence interval, respectively. The dotted vertical lines are reference lines, corresponding to corresponding to (A) 5 mmol/L, (B) 1 mmol/mol, and (C) 3 mmol/L. The top and bottom 1% of participants were omitted as outliers to provide better visualisation. Evidence for non-linearity (P_curve_) was calculated by performing a likelihood ratio test between a multivariable model with all spline terms vs. a multivariable model with only the linear term. Model adjustments include sex, ethnicity, calendar year of entry, Index of Multiple Deprivation, smoking, total comorbidities, insulin use, and diabetes duration.

**eFigure 5. The association between (A) systolic blood pressure, (B) diastolic blood pressure, (C) body mass index, and risk of dementia (adjusting for medication use) among people with type 2 diabetes in the Clinical Practice Research Datalink in England, evaluated using restricted cubic splines, and stratified by follow-up periods.**

The solid lines and shaded area represent the central risk estimate and 95% CI confidence interval, respectively. The dotted vertical lines are reference lines, corresponding to (A) 120, 130, 140, 150, 160 mmHg, (B) 70, 75, 80, 85, 90 mmHg, and (C) 20, 25, 30, 35, 40 kg/m^2^. The top and bottom 1% of participants were omitted as outliers to provide better visualisation. Evidence for non-linearity (P_curve_) was calculated by performing a likelihood ratio test between a multivariable model with all spline terms vs. a multivariable model with only the linear term. Model adjustments include sex, ethnicity, calendar year of entry, Index of Multiple Deprivation, smoking, total comorbidities, insulin use, diabetes duration, anti-hypertensive medication, lipid-lowering medication, anti-hypoglycaemic medication, and anti-platelet medication.

**eFigure 6. The association between (A) fasting plasma glucose, (B) haemoglobin A1c, and risk of dementia (adjusting for medication use) among people with type 2 diabetes in the Clinical Practice Research Datalink in England, evaluated using restricted cubic splines, and stratified by follow-up periods.**

The solid lines and shaded area represent the central risk estimate and 95% CI confidence interval, respectively. The dotted vertical lines are reference lines, corresponding to (A) 5, 6, 7, 8, 9 mmol/L and (B) 43, 48, 53, 58 mmol/mol. The top and bottom 1% of participants were omitted as outliers to provide better visualisation. Evidence for non-linearity (P_curve_) was calculated by performing a likelihood ratio test between a multivariable model with all spline terms vs. a multivariable model with only the linear term. Model adjustments include sex, ethnicity, calendar year of entry, Index of Multiple Deprivation, smoking, total comorbidities, insulin use, diabetes duration, anti-hypertensive medication, lipid-lowering medication, anti-hypoglycaemic medication, and anti-platelet medication.

**eFigure 7.** **The association between (A) total cholesterol, (B) high-density lipoprotein, (C) low-density lipoprotein, and risk of dementia (adjusting for medication use) among people with type 2 diabetes in the Clinical Practice Research Datalink in England, evaluated using restricted cubic splines, and stratified by follow-up periods.**

The solid lines and shaded area represent the central risk estimate and 95% CI confidence interval, respectively. The dotted vertical lines are reference lines, corresponding to (A) 5 mmol/L, (B) 1 mmol/mol, and (C) 3 mmol/L. The top and bottom 1% of participants were omitted as outliers to provide better visualisation. Evidence for non-linearity (P_curve_) was calculated by performing a likelihood ratio test between a multivariable model with all spline terms vs. a multivariable model with only the linear term. Model adjustments include sex, ethnicity, calendar year of entry, Index of Multiple Deprivation, smoking, total comorbidities, insulin use, diabetes duration, anti-hypertensive medication, lipid-lowering medication, anti-hypoglycaemic medication, and anti-platelet medication.

**eTable 12. The association between blood pressure, body mass index, glycaemic measures, and risk of dementia among people with type 2 diabetes in the Clinical Practice Research Datalink in England, evaluated using Cox regression, (1) including possible dementia cases and (2) adjusting for medications.**

| **Systolic blood pressure** | **<130 mmHg** | **130 – 139 mmHg** | **≥140 mmHg** |
| --- | --- | --- | --- |
| ***Main findings*** |  |  |  |
| Events/Total (per 1,000 person-years) | 5,338/63,711 (19) | 5,277/64,585 (16) | 10,914/109,575 (18) |
| Median (IQR), mmHg | 122 (116-126) | 135 (132-137) | 149 (144-157) |
| Fully adjusted, HR (95% CI) | 1.00 (Ref) | 0.80 (0.77-0.83) | 0.74 (0.72-0.77) |
| ***Including POSSIBLE DEMENTIA cases*** |  |  |  |
| Events/Total (per 1,000 person-years) | 5,376/63,691 (19) | 5,332/64,563 (16) | 11,005/109,562 (18) |
| Median (IQR), mmHg | 122 (116-126) | 135 (132-137) | 149 (144-157) |
| Fully adjusted, HR (95% CI) | 1.00 (Ref) | 0.80 (0.77-0.83) | 0.74 (0.72-0.77) |
| ***Adjusting for medications^*^*** |  |  |  |
| Events/Total (per 1,000 person-years) | 5,338/63,711 (19) | 5,277/64,585 (16) | 10,914/109,575 (18) |
| Median (IQR), mmHg | 122 (116-126) | 135 (132-137) | 149 (144-157) |
| Fully adjusted, HR (95% CI) | 1.00 (Ref) | 0.80 (0.77-0.83) | 0.76 (0.74-0.79) |
| **Diastolic blood pressure** | **<80 mmHg** | **80 – 89 mmHg** | **≥90 mmHg** |
| ***Main findings*** |  |  |  |
| Events/Total (per 1,000 person-years) | 12,974/117,938 (23) | 6,854/90,770 (14) | 1,698/29,154 (10) |
| Median (IQR), mmHg | 73 (70-77) | 83 (81-86) | 93 (91-97) |
| Fully adjusted, HR (95% CI) | 1.00 (Ref) | 1.04 (1.01-1.07) | 1.21 (1.15-1.27) |
| ***Including POSSIBLE DEMENTIA cases*** |  |  |  |
| Events/Total (per 1,000 person-years) | 13,076/117,902 (23) | 6,920/90,754 (14) | 1,714/29,151 (10) |
| Median (IQR), mmHg | 73 (70-77) | 83 (81-86) | 93 (91-97) |
| Fully adjusted, HR (95% CI) | 1.00 (Ref) | 1.04 (1.01-1.07) | 1.21 (1.15-1.27) |
| ***Adjusting for medications^*^*** |  |  |  |
| Events/Total (per 1,000 person-years) | 12,974/117,938 (23) | 6,854/90,770 (14) | 1,698/29,154 (10) |
| Median (IQR), mmHg | 73 (70-77) | 83 (81-86) | 93 (91-97) |
| Fully adjusted, HR (95% CI) | 1.00 (Ref) | 1.05 (1.02-1.08) | 1.24 (1.17-1.30) |
| **Body mass index** | **<25 kg/m^2^** | **25 – 29.9 kg/m^2^** | **≥30 kg/m^2^** |
| ***Main findings*** |  |  |  |
| Events/Total (per 1,000 person-years) | 4,861/36,241 (28) | 6,578/71,688 (17) | 6,045/98,946 (11) |
| Median (IQR), kg/m^2^ | 23 (21-24) | 28 (26-29) | 34 (32-38) |
| Fully adjusted, HR (95% CI) | 1.00 (Ref) | 0.84 (0.81-0.87) | 0.90 (0.87-0.94) |
| ***Including POSSIBLE DEMENTIA cases*** |  |  |  |
| Events/Total (per 1,000 person-years) | 4,884/36,234 (28) | 6,647/71,675 (17) | 6,114/98,924 (11) |
| Median (IQR), kg/m^2^ | 23 (21-24) | 28 (26-29) | 34 (32-38) |
| Fully adjusted, HR (95% CI) | 1.00 (Ref) | 0.84 (0.81-0.88) | 0.90 (0.87-0.94) |
| ***Adjusting for medications^*^*** |  |  |  |
| Events/Total (per 1,000 person-years) | 4,861/36,241 (28) | 6,578/71,688 (17) | 6,045/98,946 (11) |
| Median (IQR), kg/m^2^ | 23 (21-24) | 28 (26-29) | 34 (32-38) |
| Fully adjusted, HR (95% CI) | 1.00 (Ref) | 0.84 (0.81-0.88) | 0.91 (0.88-0.95) |
| **Fasting plasma glucose** | **<7.0 mmol/L** | **7.0 – 8.5 mmol/L** | **>8.5 mmol/L** |
| ***Main findings*** |  |  |  |
| Events/Total (per 1,000 person-years) | 6,214/63,155 (19) | 4,487/49,391 (16) | 6,339/70,863 (15) |
| Median (IQR), mmol/L | 6 (5-7) | 8 (7-8) | 11 (10-14) |
| Fully adjusted, HR (95% CI) | 1.00 (Ref) | 0.97 (0.93-1.01) | 1.13 (1.09-1.17) |
| ***Including POSSIBLE DEMENTIA cases*** |  |  |  |
| Events/Total (per 1,000 person-years) | 6,272/63,138 (19) | 4,536/49,382 (16) | 6,387/70,856 (16) |
| Median (IQR), mmol/L | 6 (5-7) | 8 (7-8) | 11 (10-14) |
| Fully adjusted, HR (95% CI) | 1.00 (Ref) | 0.97 (0.94-1.01) | 1.12 (1.08-1.16) |
| ***Adjusting for medications^*^*** |  |  |  |
| Events/Total (per 1,000 person-years) | 6,214/63,155 (19) | 4,487/49,391 (16) | 6,339/70,863 (15) |
| Median (IQR), mmol/L | 6 (5-7) | 8 (7-8) | 11 (10-14) |
| Fully adjusted, HR (95% CI) | 1.00 (Ref) | 0.97 (0.93-1.00) | 1.09 (1.05-1.13) |
| **Haemoglobin A1c** | **<48 mmol/mol** | **48 – 58 mmol/mol** | **>58 mmol/mol** |
| ***Main findings*** |  |  |  |
| Events/Total (per 1,000 person-years) | 5,890/58,890 (20) | 6,353/69,380 (17) | 5,078/65,818 (14) |
| Median (IQR), mmol/mol | 43 (40-45) | 52 (50-55) | 72 (64-85) |
| Fully adjusted, HR (95% CI) | 1.00 (Ref) | 0.94 (0.91-0.98) | 1.07 (1.03-1.11) |
| ***Including POSSIBLE DEMENTIA cases*** |  |  |  |
| Events/Total (per 1,000 person-years) | 5,941/58,864 (20) | 6,418/69,368 (18) | 5,119/65,808 (14) |
| Median (IQR), mmol/mol | 43 (40-45) | 52 (50-55) | 72 (64-85) |
| Fully adjusted, HR (95% CI) | 1.00 (Ref) | 0.94 (0.91-0.98) | 1.07 (1.03-1.11) |
| ***Adjusting for medications^*^*** |  |  |  |
| Events/Total (per 1,000 person-years) | 5,890/58,890 (20) | 6,353/69,380 (17) | 5,078/65,818 (14) |
| Median (IQR), mmol/mol | 43 (40-45) | 52 (50-55) | 72 (64-85) |
| Fully adjusted, HR (95% CI) | 1.00 (Ref) | 0.92 (0.88-0.95) | 0.99 (0.95-1.04) |

Age was used as the timescale for Cox regression. The fully adjusted model included sex, ethnicity, Multiple Index of Deprivation, smoking status, total comorbidities, diabetes duration and insulin use.

^*^Adjusting for medications additionally included anti-hypertensive medication, lipid-lowering medication, anti-hypoglycaemic medication, and anti-platelet medication.

CI, confidence interval; HR, hazard ratio; IQR, interquartile range.

**eTable 13. The association between total cholesterol, high-density lipoprotein, low-density lipoprotein, and risk of dementia among people with type 2 diabetes in the Clinical Practice Research Datalink in England, evaluated using Cox regression, (1) including possible dementia cases and (2) adjusting for medications.**

| **Total cholesterol** | **≤5.0 mmol/L** | **>5.0 mmol/L** |
| --- | --- | --- |
| ***Main findings*** |  |  |
| Events/Total (per 1,000 person-years) | 11,578/124,523 (18) | 6,889/86,716 (14) |
| Median (IQR), mmol/L | 4 (2-5) | 6 (5-20) |
| Fully-adjusted, HR (95% CI) | 1.00 (Ref) | 1.01 (0.97-1.04) |
| ***Including POSSIBLE DEMENTIA cases*** |  |  |
| Events/Total (per 1,000 person-years) | 11,681/124,490 (18) | 6,956/86,701 (15) |
| Median (IQR), mmol/L | 4 (2-5) | 6 (5-20) |
| Fully-adjusted, HR (95% CI) | 1.00 (Ref) | 1.00 (0.97-1.04) |
| ***Adjusting for medications^*^*** |  |  |
| Events/Total (per 1,000 person-years) | 11,578/124,523 (18) | 6,889/86,716 (14) |
| Median (IQR), mmol/L | 4 (2-5) | 6 (5-20) |
| Fully-adjusted, HR (95% CI) | 1.00 (Ref) | 1.01 (0.98-1.05) |
| **High-density lipoprotein** | **<1.0 mmol/L** | **≥1.0 mmol/L** |
| ***Main findings*** |  |  |
| Events/Total (per 1,000 person-years) | 3,034/46,708 (13) | 11,788/133,734 (17) |
| Median (IQR), mmol/L | 1 (0-1) | 1 (1-10) |
| Fully-adjusted, HR (95% CI) | 1.00 (Ref) | 0.93 (0.90-0.97) |
| ***Including POSSIBLE DEMENTIA cases*** |  |  |
| Events/Total (per 1,000 person-years) | 3,078/46,695 (13) | 8,900/107,465 (15) |
| Median (IQR), mmol/L | 1 (0-1) | 1 (1-2) |
| Fully-adjusted, HR (95% CI) | 1.00 (Ref) | 0.93 (0.89-0.97) |
| ***Adjusting for medications^*^*** |  |  |
| Events/Total (per 1,000 person-years) | 3,034/46,708 (13) | 11,788/133,734 (17) |
| Median (IQR), mmol/L | 1 (0-1) | 1 (1-10) |
| Fully-adjusted, HR (95% CI) | 1.00 (Ref) | 0.94 (0.90-0.98) |
| **Low-density lipoprotein** | **≤3.0 mmol/L** | **>3.0 mmol/L** |
| ***Main findings*** |  |  |
| Events/Total (per 1,000 person-years) | 7,823/89,917 (17) | 3,954/57,651 (13) |
| Median (IQR), mmol/L | 2 (1-3) | 4 (3-10) |
| Fully-adjusted, HR (95% CI) | 1.00 (Ref) | 1.02 (0.98-1.06) |
| ***Including POSSIBLE DEMENTIA cases*** |  |  |
| Events/Total (per 1,000 person-years) | 7,903/89,893 (17) | 3,995/57,639 (13) |
| Median (IQR), mmol/L | 2 (1-3) | 4 (3-10) |
| Fully-adjusted, HR (95% CI) | 1.00 (Ref) | 1.01 (0.98-1.05) |
| ***Adjusting for medications^*^*** |  |  |
| Events/Total (per 1,000 person-years) | 7,823/89,917 (17) | 3,954/57,651 (13) |
| Median (IQR), mmol/L | 2 (1-3) | 4 (3-10) |
| Fully-adjusted, HR (95% CI) | 1.00 (Ref) | 1.03 (0.99-1.07) |

Age was used as the timescale for Cox regression. The fully adjusted model included sex, ethnicity, Multiple Index of Deprivation, smoking status, total comorbidities, diabetes duration and insulin use.

^*^Adjusting for medications additionally included anti-hypertensive medication, lipid-lowering medication, anti-hypoglycaemic medication, and anti-platelet medication.

CI, confidence interval; HR, hazard ratio; IQR, interquartile range.

**eFigure 8. The association between (A) systolic blood pressure, (B) diastolic blood pressure, (C) body mass index, and risk of dementia among people with type 2 diabetes in the Clinical Practice Research Datalink in England, evaluated using restricted cubic splines, and stratified by follow-up periods (inverse probability weighted).**

The solid lines and shaded area represent the central risk estimate and 95% CI confidence interval, respectively. The dotted vertical lines are reference lines, corresponding to (A) 120, 130, 140, 150, 160 mmHg, (B) 70, 75, 80, 85, 90 mmHg, and (C) 20, 25, 30, 35, 40 kg/m^2^. The top and bottom 1% of participants were omitted as outliers to provide better visualisation. Model adjustments include sex, ethnicity, calendar year of entry, Index of Multiple Deprivation, smoking, total comorbidities, insulin use, and diabetes duration.

**eFigure 9. The association between (A) fasting plasma glucose, (B) haemoglobin A1c, and risk of dementia among people with type 2 diabetes in the Clinical Practice Research Datalink in England, evaluated using restricted cubic splines, and stratified by follow-up periods (inverse probability weighted).**

The solid lines and shaded area represent the central risk estimate and 95% CI confidence interval, respectively. The dotted vertical lines are reference lines, corresponding to (A) 5, 6, 7, 8, 9 mmol/L and (B) 43, 48, 53, 58 mmol/mol. The top and bottom 1% of participants were omitted as outliers to provide better visualisation. Model adjustments include sex, ethnicity, calendar year of entry, Index of Multiple Deprivation, smoking, total comorbidities, insulin use, and diabetes duration.

**eFigure 10. The association between (A) total cholesterol, (B) high-density lipoprotein, (C) low-density lipoprotein, and risk of dementia among people with type 2 diabetes in the Clinical Practice Research Datalink in England, evaluated using restricted cubic splines, and stratified by follow-up periods (inverse probability weighted).**

The solid lines and shaded area represent the central risk estimate and 95% CI confidence interval, respectively. The dotted vertical lines are reference lines, corresponding to corresponding to (A) 5 mmol/L, (B) 1 mmol/mol, and (C) 3 mmol/L. The top and bottom 1% of participants were omitted as outliers to provide better visualisation. Model adjustments include sex, ethnicity, calendar year of entry, Index of Multiple Deprivation, smoking, total comorbidities, insulin use, and diabetes duration.

**eTable 14. The association between blood pressure, body mass index, glycaemic measures, and risk of dementia among people with type 2 diabetes in the Clinical Practice Research Datalink in England, evaluated using Cox regression, stratified by follow-up periods and weighted using inverse probability weights.**

| **Systolic blood pressure** | **<130 mmHg** | **130 – 139 mmHg** | **≥140 mmHg** |
| --- | --- | --- | --- |
| ***Follow-up <5 years*** |  |  |  |
| Weighted events/Total (per 1,000 P-Y) | 2,777/37,892 (34) | 2,352/35,402 (29) | 4,652/61,108 (32) |
| Weighted median (IQR), mmHg | 121 (115-126) | 135 (132-137) | 149 (143-158) |
| Sex-adjusted, HR (95% CI) | 1.00 (Ref) | 0.78 (0.74-0.83) | 0.72 (0.69-0.76) |
| Intermediate model, HR (95% CI) | 1.00 (Ref) | 0.79 (0.74-0.83) | 0.72 (0.68-0.76) |
| Fully adjusted, HR (95% CI) | 1.00 (Ref) | 0.80 (0.75-0.84) | 0.73 (0.69-0.77) |
| ***Follow-up 5-10 years*** |  |  |  |
| Weighted events/Total (per 1,000 P-Y) | 1,511/16,777 (13) | 1,780/18,859 (13) | 3,666/33,000 (16) |
| Weighted median (IQR), mmHg | 122 (117-126) | 135 (132-137) | 149 (144-157) |
| Sex-adjusted, HR (95% CI) | 1.00 (Ref) | 0.88 (0.82-0.94) | 0.81 (0.76-0.86) |
| Intermediate model, HR (95% CI) | 1.00 (Ref) | 0.90 (0.84-0.96) | 0.81 (0.76-0.87) |
| Fully adjusted, HR (95% CI) | 1.00 (Ref) | 0.90 (0.83-0.96) | 0.82 (0.77-0.87) |
| ***Follow-up* ≥*10 years*** |  |  |  |
| Weighted events/Total (per 1,000 P-Y) | 938/7,048 (11) | 1,257/9,304 (12) | 2,898/18,480 (13) |
| Weighted median (IQR), mmHg | 123 (118-127) | 135 (132-137) | 150 (144-157) |
| Sex-adjusted, HR (95% CI) | 1.00 (Ref) | 0.83 (0.71-0.97) | 0.80 (0.70-0.91) |
| Intermediate model, HR (95% CI) | 1.00 (Ref) | 0.82 (0.70-0.96) | 0.78 (0.69-0.89) |
| Fully adjusted, HR (95% CI) | 1.00 (Ref) | 0.84 (0.72-0.99) | 0.80 (0.70-0.91) |
| **Diastolic blood pressure** | **<80 mmHg** | **80 – 89 mmHg** | **≥90 mmHg** |
| ***Follow-up <5 years*** |  |  |  |
| Weighted events/Total (per 1,000 P-Y) | 6,173/66,645 (41) | 2,884/50,316 (24) | 723/17,439 (18) |
| Weighted median (IQR), mmHg | 73 (69-77) | 83 (81-86) | 94 (91-98) |
| Sex-adjusted, HR (95% CI) | 1.00 (Ref) | 0.96 (0.91-1.01) | 1.05 (0.96-1.15) |
| Intermediate model, HR (95% CI) | 1.00 (Ref) | 0.96 (0.91-1.01) | 1.05 (0.95-1.15) |
| Fully adjusted, HR (95% CI) | 1.00 (Ref) | 0.97 (0.92-1.02) | 1.07 (0.98-1.18) |
| ***Follow-up 5-10 years*** |  |  |  |
| Weighted events/Total (per 1,000 P-Y) | 4,168/33,479 (18) | 2,240/26,743 (12) | 547/8,412 (9) |
| Weighted median (IQR), mmHg | 74 (70-77) | 83 (81-86) | 93 (91-97) |
| Sex-adjusted, HR (95% CI) | 1.00 (Ref) | 1.08 (1.03-1.14) | 1.29 (1.18-1.40) |
| Intermediate model, HR (95% CI) | 1.00 (Ref) | 1.09 (1.03-1.15) | 1.29 (1.18-1.40) |
| Fully adjusted, HR (95% CI) | 1.00 (Ref) | 1.10 (1.05-1.16) | 1.31 (1.20-1.43) |
| ***Follow-up ≥10 years*** |  |  |  |
| Weighted events/Total (per 1,000 P-Y) | 2,812/16,076 (15) | 1,876/14,202 (11) | 407/4,550 (8) |
| Weighted median (IQR), mmHg | 74 (70-77) | 84 (81-86) | 93 (91-97) |
| Sex-adjusted, HR (95% CI) | 1.00 (Ref) | 1.17 (1.05-1.29) | 1.23 (1.03-1.46) |
| Intermediate model, HR (95% CI) | 1.00 (Ref) | 1.17 (1.05-1.29) | 1.22 (1.02-1.45) |
| Fully adjusted, HR (95% CI) | 1.00 (Ref) | 1.20 (1.08-1.33) | 1.26 (1.05-1.50) |
| **Body mass index** | **<25 kg/m^2^** | **25 – 29.9 kg/m^2^** | **≥30 kg/m^2^** |
| ***Follow-up <5 years*** |  |  |  |
| Weighted events/Total (per 1,000 P-Y) | 2,567/20,819 (56) | 2,567/37,800 (29) | 2,093/53,652 (16) |
| Weighted median (IQR), kg/m^2^ | 23.0 (21.3-24.1) | 27.6 (26.4-28.8) | 34.1 (31.8-37.8) |
| Sex-adjusted, HR (95% CI) | 1.00 (Ref) | 0.76 (0.72-0.81) | 0.66 (0.61-0.70) |
| Intermediate model, HR (95% CI) | 1.00 (Ref) | 0.77 (0.72-0.82) | 0.66 (0.62-0.71) |
| Fully adjusted, HR (95% CI) | 1.00 (Ref) | 0.78 (0.73-0.83) | 0.67 (0.62-0.72) |
| ***Follow-up 5-10 years*** |  |  |  |
| Weighted events/Total (per 1,000 P-Y) | 1,475/10,044 (21) | 2,295/21,868 (15) | 2,254/30,021 (11) |
| Weighted median (IQR), kg/m^2^ | 23.2 (21.7-24.2) | 27.6 (26.4-28.8) | 34 (31.8-37.6) |
| Sex-adjusted, HR (95% CI) | 1.00 (Ref) | 0.95 (0.89-1.02) | 1.13 (1.05-1.21) |
| Intermediate model, HR (95% CI) | 1.00 (Ref) | 0.97 (0.91-1.04) | 1.15 (1.07-1.23) |
| Fully adjusted, HR (95% CI) | 1.00 (Ref) | 0.98 (0.91-1.04) | 1.16 (1.08-1.24) |
| ***Follow-up ≥10 years*** |  |  |  |
| Weighted events/Total (per 1,000 P-Y) | 948/4,946 (16) | 1,949/12,454 (13) | 1,793/15,271 (10) |
| Weighted median (IQR), kg/m^2^ | 23.4 (22.0-24.3) | 27.6 (26.3-28.7) | 33.8 (31.6-37.4) |
| Sex-adjusted, HR (95% CI) | 1.00 (Ref) | 1.01 (0.88-1.16) | 1.25 (1.09-1.44) |
| Intermediate model, HR (95% CI) | 1.00 (Ref) | 1.03 (0.90-1.18) | 1.29 (1.12-1.48) |
| Fully adjusted, HR (95% CI) | 1.00 (Ref) | 1.03 (0.90-1.18) | 1.30 (1.13-1.49) |
| **Fasting plasma glucose** | **<7.0 mmol/L** | **7.0 – 8.5 mmol/L** | **>8.5 mmol/L** |
| ***Follow-up <5 years*** |  |  |  |
| Weighted events/Total (per 1,000 P-Y) | 2,683/32,282 (35) | 1,708/24,664 (28) | 2,686/38,226 (29) |
| Weighted median (IQR), mmol/L | 5.9 (5.2-6.5) | 7.6 (7.3-8.0) | 11.5 (9.7-14.5) |
| Sex-adjusted, HR (95% CI) | 1.00 (Ref) | 0.91 (0.85-0.97) | 1.07 (1.00-1.13) |
| Intermediate model, HR (95% CI) | 1.00 (Ref) | 0.91 (0.85-0.97) | 1.05 (0.99-1.12) |
| Fully adjusted, HR (95% CI) | 1.00 (Ref) | 0.91 (0.86-0.98) | 1.03 (0.97-1.09) |
| ***Follow-up 5-10 years*** |  |  |  |
| Weighted events/Total (per 1,000 P-Y) | 2,116/19,670 (15) | 1,584/16,191 (14) | 2,075/22,020 (13) |
| Weighted median (IQR), mmol/L | 6.0 (5.2-6.5) | 7.6 (7.3-8.0) | 11.4 (9.6-14.4) |
| Sex-adjusted, HR (95% CI) | 1.00 (Ref) | 0.98 (0.92-1.05) | 1.14 (1.07-1.21) |
| Intermediate model, HR (95% CI) | 1.00 (Ref) | 0.99 (0.93-1.06) | 1.14 (1.07-1.21) |
| Fully adjusted, HR (95% CI) | 1.00 (Ref) | 1.00 (0.93-1.06) | 1.11 (1.04-1.18) |
| ***Follow-up ≥10 years*** |  |  |  |
| Weighted events/Total (per 1,000 P-Y) | 1,331/8,983 (13) | 1,273/8,829 (12) | 1,717/12,544 (12) |
| Weighted median (IQR), mmol/L | 6.1 (5.4-6.6) | 7.6 (7.3-8.0) | 11.4 (9.6-14.2) |
| Sex-adjusted, HR (95% CI) | 1.00 (Ref) | 1.10 (0.96-1.26) | 1.27 (1.12-1.44) |
| Intermediate model, HR (95% CI) | 1.00 (Ref) | 1.10 (0.97-1.26) | 1.25 (1.10-1.41) |
| Fully adjusted, HR (95% CI) | 1.00 (Ref) | 1.12 (0.98-1.27) | 1.24 (1.10-1.41) |
| **Haemoglobin A1c** | **<48 mmol/mol** | **48 – 58 mmol/mol** | **>58 mmol/mol** |
| ***Follow-up <5 years*** |  |  |  |
| Weighted events/Total (per 1,000 P-Y) | 2,749/32,293 (37) | 2,674/37,694 (30) | 2,176/37,772 (25) |
| Weighted median (IQR), mmol/mol | 43 (39-45) | 52 (50-55) | 72 (64-86) |
| Sex-adjusted, HR (95% CI) | 1.00 (Ref) | 0.90 (0.84-0.95) | 0.99 (0.93-1.06) |
| Intermediate model, HR (95% CI) | 1.00 (Ref) | 0.89 (0.84-0.95) | 0.98 (0.92-1.04) |
| Fully adjusted, HR (95% CI) | 1.00 (Ref) | 0.88 (0.83-0.94) | 0.93 (0.87-0.99) |
| ***Follow-up 5-10 years*** |  |  |  |
| Weighted events/Total (per 1,000 P-Y) | 1,833/16,338 (16) | 1,984/19,629 (14) | 1,738/19,304 (13) |
| Weighted median (IQR), mmol/mol | 43 (40-45) | 52 (50-55) | 72 (64-84) |
| Sex-adjusted, HR (95% CI) | 1.00 (Ref) | 0.98 (0.92-1.05) | 1.22 (1.14-1.30) |
| Intermediate model, HR (95% CI) | 1.00 (Ref) | 0.97 (0.91-1.03) | 1.20 (1.12-1.28) |
| Fully adjusted, HR (95% CI) | 1.00 (Ref) | 0.96 (0.90-1.02) | 1.15 (1.07-1.23) |
| ***Follow-up ≥10 years*** |  |  |  |
| Weighted events/Total (per 1,000 P-Y) | 1,371/9,008 (13) | 1,797/11,248 (14) | 1,458/10,802 (11) |
| Weighted median (IQR), mmol/mol | 42 (40-44) | 52 (50-55) | 70 (64-83) |
| Sex-adjusted, HR (95% CI) | 1.00 (Ref) | 1.09 (0.97-1.23) | 1.35 (1.18-1.55) |
| Intermediate model, HR (95% CI) | 1.00 (Ref) | 1.07 (0.95-1.20) | 1.32 (1.16-1.51) |
| Fully adjusted, HR (95% CI) | 1.00 (Ref) | 1.06 (0.94-1.19) | 1.26 (1.10-1.45) |

Inverse probability was calculated as 1/the odds (logistic regression, adjusted for multiple covariates) of being included in sub-cohort, which was applied as a weighting to the analysis. Age was used as the timescale for Cox regression. The intermediate model adjusted for sex, ethnicity, Multiple Index of Deprivation, smoking status, and total comorbidities. The fully adjusted model also additionally included diabetes duration and insulin use. CI, confidence interval; HR, hazard ratio; IQR, interquartile range; P-Y, person-years.

**eTable 15. The association between total cholesterol, high-density lipoprotein, and low-density lipoprotein, and risk of dementia among people with type 2 diabetes in the Clinical Practice Research Datalink in England, evaluated using Cox regression, stratified by follow-up periods and weighted using inverse probability weights.**

| **Total cholesterol** | **≤5.0 mmol/L** | **>5.0 mmol/L** |
| --- | --- | --- |
| ***Follow-up <5 years*** |  |  |
| Weighted events/Total (per 1,000 P-Y) | 4,955/67,391 (31) | 2,901/47,771 (26) |
| Weighted median (IQR), mmol/L | 4.2 (3.7-4.6) | 5.8 (5.4-6.4) |
| Sex-adjusted, HR (95% CI) | 1.00 (Ref) | 0.99 (0.94-1.04) |
| Intermediate model, HR (95% CI) | 1.00 (Ref) | 0.98 (0.93-1.04) |
| Fully-adjusted, HR (95% CI) | 1.00 (Ref) | 1.01 (0.96-1.07) |
| ***Follow-up 5-10 years*** |  |  |
| Weighted events/Total (per 1,000 P-Y) | 3,830/37,096 (15) | 2,369/25,803 (13) |
| Weighted median (IQR), mmol/L | 4.3 (3.8-4.7) | 5.7 (5.4-6.3) |
| Sex-adjusted, HR (95% CI) | 1.00 (Ref) | 0.99 (0.94-1.05) |
| Intermediate model, HR (95% CI) | 1.00 (Ref) | 1.00 (0.95-1.06) |
| Fully-adjusted, HR (95% CI) | 1.00 (Ref) | 1.01 (0.96-1.07) |
| ***Follow-up ≥10 years*** |  |  |
| Weighted events/Total (per 1,000 P-Y) | 3,018/19,426 (13) | 1,762/13,752 (11) |
| Weighted median (IQR), mmol/L | 4.3 (3.8-4.7) | 5.7 (5.3-6.3) |
| Sex-adjusted, HR (95% CI) | 1.00 (Ref) | 0.88 (0.79-0.97) |
| Intermediate model, HR (95% CI) | 1.00 (Ref) | 0.87 (0.79-0.96) |
| Fully-adjusted, HR (95% CI) | 1.00 (Ref) | 0.89 (0.80-0.99) |
| **High-density lipoprotein** | **<1.0 mmol/L** | **≥1.0 mmol/L** |
| ***Follow-up <5 years*** |  |  |
| Weighted events/Total (per 1,000 P-Y) | 1,354/26,839 (22) | 5,058/73,206 (29) |
| Weighted median (IQR), mmol/L | 0.9 (0.8-1.0) | 1.3 (1.2-1.6) |
| Sex-adjusted, HR (95% CI) | 1.00 (Ref) | 0.94 (0.88-1.01) |
| Intermediate model, HR (95% CI) | 1.00 (Ref) | 0.96 (0.89-1.03) |
| Fully-adjusted, HR (95% CI) | 1.00 (Ref) | 0.96 (0.90-1.03) |
| ***Follow-up 5-10 years*** |  |  |
| Weighted events/Total (per 1,000 P-Y) | 1,044/13,909 (11) | 3,993/39,232 (14) |
| Weighted median (IQR), mmol/L | 0.9 (0.8-1.0) | 1.3 (1.2-1.5) |
| Sex-adjusted, HR (95% CI) | 1.00 (Ref) | 0.92 (0.86-0.99) |
| Intermediate model, HR (95% CI) | 1.00 (Ref) | 0.93 (0.87-1.00) |
| Fully-adjusted, HR (95% CI) | 1.00 (Ref) | 0.94 (0.87-1.01) |
| ***Follow-up ≥10 years*** |  |  |
| Weighted events/Total (per 1,000 P-Y) | 766/6,903 (10) | 2,893/20,353 (12) |
| Weighted median (IQR), mmol/L | 0.9 (0.8-1.0) | 1.3 (1.2-1.6) |
| Sex-adjusted, HR (95% CI) | 1.00 (Ref) | 0.85 (0.74-0.98) |
| Intermediate model, HR (95% CI) | 1.00 (Ref) | 0.84 (0.73-0.97) |
| Fully-adjusted, HR (95% CI) | 1.00 (Ref) | 0.85 (0.74-0.98) |
| **Low-density lipoprotein** | **≤3.0 mmol/L** | **>3.0 mmol/L** |
| ***Follow-up <5 years*** |  |  |
| Weighted events/Total (per 1,000 P-Y) | 3,381/49,944 (29) | 1,723/32,356 (23) |
| Weighted median (IQR), mmol/L | 2.3 (1.8-2.6) | 3.7 (3.3-4.2) |
| Sex-adjusted, HR (95% CI) | 1.00 (Ref) | 1.03 (0.96-1.10) |
| Intermediate model, HR (95% CI) | 1.00 (Ref) | 1.03 (0.97-1.11) |
| Fully-adjusted, HR (95% CI) | 1.00 (Ref) | 1.06 (0.99-1.14) |
| ***Follow-up 5-10 years*** |  |  |
| Weighted events/Total (per 1,000 P-Y) | 2,642/27,474 (14) | 1,367/16,537 (12) |
| Weighted median (IQR), mmol/L | 2.3 (1.8-2.6) | 3.6 (3.3-4.1) |
| Sex-adjusted, HR (95% CI) | 1.00 (Ref) | 1.00 (0.93-1.06) |
| Intermediate model, HR (95% CI) | 1.00 (Ref) | 1.01 (0.95-1.08) |
| Fully-adjusted, HR (95% CI) | 1.00 (Ref) | 1.03 (0.96-1.10) |
| ***Follow-up ≥10 years*** |  |  |
| Weighted events/Total (per 1,000 P-Y) | 1,880/13,341 (12) | 961/7,916 (10) |
| Weighted median (IQR), mmol/L | 2.3 (1.9-2.7) | 3.6 (3.3-4.1) |
| Sex-adjusted, HR (95% CI) | 1.00 (Ref) | 0.89 (0.77-1.03) |
| Intermediate model, HR (95% CI) | 1.00 (Ref) | 0.88 (0.76-1.02) |
| Fully-adjusted, HR (95% CI) | 1.00 (Ref) | 0.89 (0.77-1.03) |

Age was used as the timescale for Cox regression. The intermediate model adjusted for sex, ethnicity, Multiple Index of Deprivation, smoking status, and total comorbidities. The fully adjusted model also additionally included diabetes duration and insulin use. CI, confidence interval; HR, hazard ratio; IQR, interquartile range; P-Y, person-years.

**eTable 16. The association between blood pressure, body mass index, glycaemic measures, and risk of dementia among people with type 2 diabetes in the Clinical Practice Research Datalink in England, evaluated using Cox regression and a modified Fine and Gray model.**

| **Cardiometabolic factor** | **Group 1** | **Group 2** | **Group 3** |
| --- | --- | --- | --- |
| **Systolic blood pressure** | **<130 mmHg** | **130 – 139 mmHg** | **≥140 mmHg** |
| Events/Total (per 1,000 person-years) | 5,338/63,711 (19) | 5,277/64,585 (16) | 10,914/109,575 (18) |
| Median (IQR), mmHg | 122 (116-126) | 135 (132-137) | 149 (144-157) |
| Cox Regression, HR (95% CI) | 1.00 (Ref) | 0.80 (0.77-0.83) | 0.74 (0.72-0.77) |
| Fine and Gray, HR (95% CI) | 1.00 (Ref) | 0.86 (0.83-0.89) | 0.82 (0.79-0.85) |
| **Diastolic blood pressure** | **<80 mmHg** | **80 – 89 mmHg** | **≥90 mmHg** |
| Events/Total (per 1,000 person-years) | 12,974/117,938 (23) | 6,854/90,770 (14) | 1,698/29,154 (10) |
| Median (IQR), mmHg | 73 (70-77) | 83 (81-86) | 93 (91-97) |
| Cox Regression, HR (95% CI) | 1.00 (Ref) | 1.04 (1.01-1.07) | 1.21 (1.15-1.27) |
| Fine and Gray, HR (95% CI) | 1.00 (Ref) | 1.04 (1.01-1.07) | 1.18 (1.12-1.24) |
| **Body mass index** | **<25 kg/m^2^** | **25 – 29.9 kg/m^2^** | **≥30 kg/m^2^** |
| Events/Total (per 1,000 person-years) | 4,861/36,241 (28) | 6,578/71,688 (17) | 6,045/98,946 (11) |
| Median (IQR), mmHg | 23 (21-24) | 28 (26-29) | 34 (32-38) |
| Cox Regression, HR (95% CI) | 1.00 (Ref) | 0.84 (0.81-0.87) | 0.90 (0.87-0.94) |
| Fine and Gray, HR (95% CI) | 1.00 (Ref) | 0.86 (0.83-0.90) | 0.89 (0.86-0.93) |
| **Fasting plasma glucose** | **<7.0 mmol/L** | **7.0 – 8.5 mmol/L** | **>8.5 mmol/L** |
| Events/Total (per 1,000 person-years) | 6,214/63,155 (19) | 4,487/49,391 (16) | 6,339/70,863 (15) |
| Median (IQR), mmol/L | 6 (5-7) | 8 (7-8) | 11 (10-14) |
| Cox Regression, HR (95% CI) | 1.00 (Ref) | 0.97 (0.93-1.01) | 1.13 (1.09-1.17) |
| Fine and Gray, HR (95% CI) | 1.00 (Ref) | 0.96 (0.93-1.00) | 1.07 (1.04-1.11) |
| **Haemoglobin A1c** | **<48 mmol/mol** | **48 – 58 mmol/mol** | **>58 mmol/mol** |
| Events/Total (per 1,000 person-years) | 5,890/58,890 (20) | 6,353/69,380 (17) | 5,078/65,818 (14) |
| Median (IQR), mmol/mol | 43 (40-45) | 52 (50-55) | 72 (64-85) |
| Cox Regression, HR (95% CI) | 1.00 (Ref) | 0.94 (0.91-0.98) | 1.07 (1.03-1.11) |
| Fine and Gray, HR (95% CI) | 1.00 (Ref) | 0.94 (0.91-0.98) | 1.02 (0.98-1.06) |

Age was used as the timescale for both models. Adjustments include sex, ethnicity, Multiple Index of Deprivation, smoking status, total comorbidities, diabetes duration and insulin use. CI, confidence interval; HR, hazard ratio; IQR, interquartile range.

**eTable 17. The association between total cholesterol, high-density lipoprotein, and low-density lipoprotein, and risk of dementia among people with type 2 diabetes in the Clinical Practice Research Datalink in England, evaluated using Cox regression and a modified Fine and Gray model.**

| **Cardiometabolic Factor** | **Group 1** | **Group 2** |
| --- | --- | --- |
| **Total cholesterol** | **≤5.0 mmol/L** | **>5.0 mmol/L** |
| Events/Total (per 1,000 person-years) | 11,578/124,523 (18) | 6,889/86,716 (14) |
| Median (IQR), mmol/L | 4 (4-5) | 6 (5-6) |
| Cox Regression, HR (95% CI) | 1.00 (Ref) | 1.01 (0.97-1.04) |
| Fine and Gray, HR (95% CI) | 1.00 (Ref) | 1.03 (1.00-1.06) |
| **High-density lipoprotein** | **<1.0 mmol/L** | **≥1.0 mmol/L** |
| Events/Total (per 1,000 person-years) | 3,034/46,708 (13) | 11,788/133,734 (17) |
| Median (IQR), mmol/L | 4 (4-5) | 6 (5-6) |
| Cox Regression, HR (95% CI) | 1.00 (Ref) | 0.93 (0.90-0.97) |
| Fine and Gray, HR (95% CI) | 1.00 (Ref) | 0.99 (0.95-1.03) |
| **Low-density lipoprotein** | **≤3.0 mmol/L** | **>3.0 mmol/L** |
| Events/Total (per 1,000 person-years) | 7,823/89,917 (17) | 3,954/57,651 (13) |
| Median (IQR), mmol/L | 4 (4-5) | 6 (5-6) |
| Cox Regression, HR (95% CI) | 1.00 (Ref) | 1.02 (0.98-1.06) |
| Fine and Gray, HR (95% CI) | 1.00 (Ref) | 1.04 (1.00-1.08) |

Age was used as the timescale for both models. Adjustments include sex, ethnicity, Multiple Index of Deprivation, smoking status, total comorbidities, diabetes duration and insulin use. CI, confidence interval; HR, hazard ratio; IQR, interquartile range.

**eTable 18. The association between blood pressure, body mass index, glycaemic measures, and risk of dementia among people with type 2 diabetes and more than 10 years of** **follow-up in the Clinical Practice Research Datalink in England, evaluated using Cox regression and a modified Fine and Gray model.**

| **Cardiometabolic factor** | **Group 1** | **Group 2** | **Group 3** |
| --- | --- | --- | --- |
| **Systolic blood pressure** | **<130 mmHg** | **130 – 139 mmHg** | **≥140 mmHg** |
| Events/Total (per 1,000 person-years) | 742/6,767 (9) | 1,082/8,863 (10) | 2,852/19,202 (12) |
| Median (IQR), mmHg | 122 (118-127) | 135 (132-137) | 150 (144-158) |
| Cox Regression, HR (95% CI) | 1.00 (Ref) | 0.86 (0.78-0.94) | 0.80 (0.74-0.87) |
| Fine and Gray, HR (95% CI) | 1.00 (Ref) | 0.86 (0.78-0.94) | 0.80 (0.74-0.87) |
| **Diastolic blood pressure** | **<80 mmHg** | **80 – 89 mmHg** | **≥90 mmHg** |
| Events/Total (per 1,000 person-years) | 2,329/14,356 (23) | 1,861/15,164 (14) | 486/5,308 (10) |
| Median (IQR), mmHg | 74 (70-77) | 84 (81-86) | 93 (91-97) |
| Cox Regression, HR (95% CI) | 1.00 (Ref) | 1.19 (1.12-1.27) | 1.37 (1.24-1.51) |
| Fine and Gray, HR (95% CI) | 1.00 (Ref) | 1.17 (1.10-1.24) | 1.38 (1.25-1.52) |
| **Body mass index** | **<25 kg/m^2^** | **25 – 29.9 kg/m^2^** | **≥30 kg/m^2^** |
| Events/Total (per 1,000 person-years) | 821/4,751 (14) | 1,756/12,281 (11) | 1,762/15,639 (9) |
| Median (IQR), kg/m^2^ | 23 (22-24) | 28 (26-29) | 34 (32-37) |
| Cox Regression, HR (95% CI) | 1.00 (Ref) | 1.07 (0.98-1.16) | 1.43 (1.31-1.56) |
| Fine and Gray, HR (95% CI) | 1.00 (Ref) | 1.04 (0.96-1.12) | 1.34 (1.24-1.46) |
| **Fasting plasma glucose** | **<7.0 mmol/L** | **7.0 – 8.5 mmol/L** | **>8.5 mmol/L** |
| Events/Total (per 1,000 person-years) | 1,202/8,594 (19) | 1,136/8,332 (16) | 1,687/13,430 (15) |
| Median (IQR), mmol/L | 6 (6-7) | 8 (7-8) | 11 (10-14) |
| Cox Regression, HR (95% CI) | 1.00 (Ref) | 1.11 (1.02-1.20) | 1.24 (1.15-1.33) |
| Fine and Gray, HR (95% CI) | 1.00 (Ref) | 1.11 (1.03-1.20) | 1.24 (1.15-1.33) |
| **Haemoglobin A1c** | **<48 mmol/mol** | **48 – 58 mmol/mol** | **>58 mmol/mol** |
| Events/Total (per 1,000 person-years) | 1,211/8,643 (20) | 1,682/11,259 (17) | 1,308/11,156 (14) |
| Median (IQR), mmol/mol | 42 (38-44) | 53 (50-55) | 69 (64-81) |
| Cox Regression, HR (95% CI) | 1.00 (Ref) | 1.07 (1.00-1.16) | 1.25 (1.16-1.36) |
| Fine and Gray, HR (95% CI) | 1.00 (Ref) | 1.06 (0.98-1.14) | 1.24 (1.15-1.34) |

Age was used as the timescale for both models. Adjustments include sex, ethnicity, Multiple Index of Deprivation, smoking status, total comorbidities, diabetes duration and insulin use. CI, confidence interval; HR, hazard ratio; IQR, interquartile range.

**eTable 19. The association between total cholesterol, high-density lipoprotein, and low-density lipoprotein, and risk of dementia among people with type 2 diabetes and more than 10 years of** **follow-up in the Clinical Practice Research Datalink in England, evaluated using Cox regression and a modified Fine and Gray model.**

| **Cardiometabolic Factor** | **Group 1** | **Group 2** |
| --- | --- | --- |
| **Total cholesterol** | **≤5.0 mmol/L** | **>5.0 mmol/L** |
| Events/Total (per 1,000 person-years) | 2,601/18,548 (11) | 1,800/14,630 (10) |
| Median (IQR), mmol/L | 4 (4-5) | 6 (5-6) |
| Cox Regression, HR (95% CI) | 1.00 (Ref) | 0.96 (0.90-1.02) |
| Fine and Gray, HR (95% CI) | 1.00 (Ref) | 0.97 (0.92-1.03) |
| **High-density lipoprotein** | **<1.0 mmol/L** | **≥1.0 mmol/L** |
| Events/Total (per 1,000 person-years) | 660/6,359 (8) | 2,775/20,897 (11) |
| Median (IQR), mmol/L | 1 (1-1) | 1 (1-2) |
| Cox Regression, HR (95% CI) | 1.00 (Ref) | 0.88 (0.80-0.96) |
| Fine and Gray, HR (95% CI) | 1.00 (Ref) | 0.90 (0.82-0.98) |
| **Low-density lipoprotein** | **≤3.0 mmol/L** | **>3.0 mmol/L** |
| Events/Total (per 1,000 person-years) | 1,731/12,857 (11) | 1,006/8,400 (10) |
| Median (IQR), mmol/L | 2 (2-3) | 4 (3-4) |
| Cox Regression, HR (95% CI) | 1.00 (Ref) | 0.95 (0.88-1.03) |
| Fine and Gray, HR (95% CI) | 1.00 (Ref) | 0.97 (0.90-1.05) |

Age was used as the timescale for both models. Adjustments include sex, ethnicity, Multiple Index of Deprivation, smoking status, total comorbidities, diabetes duration and insulin use. CI, confidence interval; HR, hazard ratio; IQR, interquartile range.

**REFERENCES**

1.	United Kingdom Ministry of Housing CLG. National Statistics: English indices of deprivation 2015. 2015. <https://www.gov.uk/government/statistics/english-indices-of-deprivation-2015>. Accessed 03 Feb 2022.

2.	Herrett E, Gallagher AM, Bhaskaran K, et al. Data Resource Profile: Clinical Practice Research Datalink (CPRD). International Journal of Epidemiology. 2015;44(3):827-36. doi:10.1093/ije/dyv098
